# Targeting PDCD4 in cancer and atrial fibrillation: mechanistic insights from integrated multi-omics and single-cell analysis

**DOI:** 10.3389/fonc.2025.1593815

**Published:** 2025-07-22

**Authors:** Juledezi Hailati, Zhiqiang Liu, Lei Zhang, Muhuyati Wulasihan

**Affiliations:** Cardiovascular Disease Center, The First Affiliated Hospital of Xinjiang Medical University, Urumqi, China

**Keywords:** atrial fibrillation, PDCD4, transcriptome sequencing, single-cell RNA sequencing, pan-cancer analysis

## Abstract

**Background:**

Atrial fibrillation (AF) is a complicated and varied cardiovascular disorder with inadequate understanding of its molecular underpinnings. While Programmed cell death factor 4 (PDCD4) has been associated in several illnesses, its particular significance in AF remains unknown. This work seeks to discover PDCD4-associated critical genes and clarify their regulation processes.

**Method:**

We built a protein-protein interaction (PPI) network to emphasize important biological interactions and used transcriptome analysis to find differentially expressed genes (DEGs). Regulatory mechanisms were explored through miRNA-mRNA and transcription factor (TF) analysis. Single-cell RNA sequencing (SCRNA-SEQ) data were utilized to identify crucial cell types and intercellular communication patterns associated with key genes.

**Results:**

qRT-PCR analysis of peripheral blood mononuclear cells (PBMCs) from AF patients and healthy controls revealed a significant upregulation of PDCD4 in AF patients. Through differential expression analysis and PPI network construction, 11 key genes were identified. In addition, mmu-miR-429-3p regulates Sirt1 while Wt1 shares regulatory roles with PDCD4, Wasl, and Abl2, and that Sirt1 and Atad5 are both regulated by Thap9. Drug prediction analyses revealed sirtinol and trichostatin as promising therapeutic drugs for targeting Atad5 and Sirt1, respectively, with good molecular docking scores (< -5 kcal/mol). SCRNA-SEQ data pinpointed arterial and venous endothelial cells as critical cell types associated with the key genes. Finally, we also found that PDCD4 dysregulation in cancers like ACC may increase AF risk through immune modulation, suggesting that targeting PDCD4 could benefit both AF and ACC patients.

**Conclusions:**

This study demonstrates that PDCD4 modulates AF progression by regulating key genes and pathways involved in inflammation, fibrosis, and metabolic processes. Insights from transcriptome and single-cell analysis give a full knowledge of the molecular processes underlying AF and indicate PDCD4 as a possible therapeutic target.

## 1 Introduction

Atrial fibrillation (AF), the most prevalent cardiac arrhythmia worldwide, is a major contributor to morbidity, mortality, and reduced quality of life related to cardiovascular disease (1). There is a significant public health concern around AF, as it affects over 37.5 million people globally and is expected to increase significantly in the coming 30 years (2). Risk factors that have long been recognized include becoming older, being overweight, having hypertension, diabetes, sleep apnea, having a history of myocardial infarction or heart failure, smoking, and having a genetic predisposition (3). Although AF seldom causes death on its own, it is associated with several major health issues, including renal failure, cancer, heart attacks, strokes, dementia, blood clots, heart attacks, and chronic kidney disease (4). Current treatment options have their limits, as evidenced by significant recurrence rates and residual stroke risk, especially with anticoagulation, despite advances in pharmacological and catheter-based therapies (5). In order to overcome these obstacles and develop better prognostic and therapeutic methods, we must have a better knowledge of the fundamental processes of AF.

An extensively expressed protein first identified for its role in apoptosis (6), PDCD4 goes by several other names, including H731, TIS, 195/15a, and MA-3. PDCD4 is a newly identified tumor suppressor that induces apoptosis and suppresses cell proliferation, invasion, and metastasis (7). New research suggests that PDCD4, in addition to its known functions in cancer, regulates oxidative stress, inflammation, and metabolic pathways (8), which might put it at the root of a number of metabolic illnesses. These include diabetes, obesity, polycystic ovarian syndrome, and obesity. In addition, the role of PDCD4 in cardiovascular illnesses such atherosclerosis and acute myocardial infarction has been more and more acknowledged in recent years (9, 10). In the setting of AF, fibrosis-characterized structural remodeling is an indicator of disease progression (11). New research indicates that atrial myocytes are more prone to inflammation and fibrosis when PDCD4 is expressed through the PPAR-γ/NF-κB signaling pathway (12). Nevertheless, there is still much we don’t know about how PDCD4 affects AF etiology, which is both a significant knowledge gap and a possible opportunity for therapy.

In addition to its cardiovascular functions, PDCD4 plays a critical role in tumorigenesis and cancer progression. As cancer incidence continues to rise globally—particularly among younger populations—this disease remains a major public health burden, with over 2 million new cases and more than 600,000 deaths projected in the United States alone in 2024 (13). Although cancer mortality has declined over recent decades due to earlier detection and improved therapies, the overall burden remains substantial and unevenly distributed across populations. Traditional approaches to cancer therapy, such as surgery, chemotherapy, and radiotherapy, have gradually been complemented by targeted therapies and immunotherapy, including immune checkpoint inhibitors and personalized vaccines (14). However, limitations such as drug resistance, immune evasion, and systemic toxicity persist. In this context, increasing attention has been paid to strategies that target not only cancer cells directly but also the tumor microenvironment (TME), where stromal and immune cells collectively influence tumor behavior and treatment response (15). PDCD4 has been implicated in several of these mechanisms, including its regulation by miRNAs and ubiquitin-mediated degradation, which affects its tumor suppressive activity across multiple cancers (16). These findings raise the possibility that PDCD4 may serve as a molecular nexus between tumor-intrinsic signaling and microenvironmental regulation. Therefore, investigating the pan-cancer expression, prognostic value, and immunological relevance of PDCD4 may provide broader insight into its therapeutic potential beyond atrial fibrillation.

RNA-seq, or whole-transcriptome analysis, has recently become a potent method for revealing disease-related DEGs and understanding gene expression patterns (17). Our knowledge of disease processes, biomarkers, and potential treatment targets has been greatly enhanced by this technology. Researchers studying AF have found that RNA-seq is a great tool for elucidating the molecular and genetic mechanisms that contribute to the disease’s development. We may better understand the complex molecular and cellular interactions that cause AF by combining transcriptome analysis with single-cell RNA sequencing (SCRNA-SEQ). This will allow us to identify important regulatory networks and cellular heterogeneity.

This work utilizes extensive transcriptome and cellular analysis to explore the function of PDCD4 in the development of AF. To discover important genes, pathways, and regulatory networks related to PDCD4, we used RNA sequencing in mouse models of PDCD4 deletion and overexpression. Through the integration of data from single-cell RNA sequencing, we investigated expression unique to cell types and relationships between cells. We have discovered new information about PDCD4 and its role in the evolution of AF. This suggests that PDCD4 might be a key regulator and possible target for therapy.

## 2 Methodology

### 2.1 qRT-PCR analysis of PDCD4 expression in PBMCs

This study was approved by the Ethics Committee, and informed consent was obtained from all participants. Peripheral blood samples were collected from 5 AF patients and 5 healthy controls at the First Affiliated Hospital of Xinjiang Medical University (December 2023–January 2024). Peripheral blood mononuclear cells (PBMCs) were isolated using standard protocols, and total RNA was extracted with the FastPure Cell/Tissue Total RNA Isolation Kit (Vazyme). cDNA was synthesized using the ReverTra Ace qPCR RT Master Mix with gDNA Remover. qRT-PCR was performed using SYBR Premix Ex Taq II on a real-time PCR detection system, with GAPDH as the endogenous control. The 2^(-ΔΔCt) method was used for relative gene expression analysis. Primer sequences are provided in **Supplementary Table 1**.

### 2.2 Animal models

To evaluate the function of PDCD4 in AF, this research used two mouse models: one with overexpression of PDCD4 (PDCD4-OE) and one without it (PDCD4-KO). As a control group, we utilized C57BL/6J wild-type mice. A transgenic method was used to produce PDCD4-OE mice, whereas CRISPR-Cas9 gene editing was used to produce PDCD4-KO mice. The institutional animal care and use committee’s requirements were followed in all animal procedures. All investigations were carried out on 8–10 week old mice that were kept in a controlled environment with a 12-hour light/dark cycle.

We used a mix of electrical stimulation and rapid atrial pacing (RAP) to produce AF. To summarize, isoflurane was used to put the mice to sleep, then a microelectrode was placed into the right atrium for RAP. To induce AF, a pacing rate of 450 beats per minute was applied for 1 hour. Using electrocardiography, the frequency and duration of atrial fibrillation were tracked. After AF was induced, the mice were kept for 24 hours before molecular examination of their tissue samples.

### 2.3 RNA sequencing

Total RNA was isolated from the atrial tissues of PDCD4-KO, PDCD4-OE, and wild-type control mice utilizing TRIzol reagent in accordance with the manufacturer’s instructions. The integrity of RNA was evaluated with an Agilent 2100 Bioanalyzer. High-quality RNA was utilized to construct RNA sequencing libraries with the NEBNext Ultra II RNA Library Prep Kit. The libraries were sequenced using an Illumina NovaSeq 6000 platform to produce 150-bp paired-end reads. Raw sequencing data were subjected to quality control via FastQC and subsequently trimmed using Trim Galore. All reagents were obtained from the USA unless stated differently.

### 2.4 Data collection

We used the Gene Expression Omnibus database to get the SCRNA-SEQ dataset GSE197518. Four animals with atrial fibrillation and four control mice had non-cardiomyocyte samples collected from the left atrium and included in this collection. Key cell clusters were identified, cell-cell communication was analyzed, and pseudotime was calculated using the data. These investigations sought to delve into the AF molecular landscape and the dynamics of important cell populations that have a role in the development of the illness.

### 2.5 Whole transcriptome data quality control

Transcripts per million were calculated from raw count values that were quantified using FeatureCounts software for mRNA analysis. By following these steps, we were able to compare total expression levels among samples and evaluate the dispersion of gene expression within each. Next, all transcriptome samples were subjected to principal component analysis (PC) in order to detect any possible outliers and evaluate the overall data quality.

First, the transcriptome data were analyzed for long non-coding RNAs (LncRNAs) by evaluating the raw sequencing data for quality using FastQC. Next Generation Sequencing Quality Control methods were used to filter out low-quality reads, resulting in clean data. The GRCm38.102 reference genome was used to align the clean reads with TopHat2 (v 2.1.1) (18), and Cufflinks and Cuffmerge were used to undertake transcript assembly. Annotation of LNC-RNA was based on sources such as LncRBase and The Atlas of Noncoding RNAs in Cancer (TANRIC). Using RNA-Seq by Expectation Maximization, we assessed gene and transcript expression levels. To normalize the expression data, we used FK-MMR, which stands for fragments per kilobase of transcript per million mapped reads. The ggplot2 software (v 3.3.6) (19) was used to construct box plots, and PC was applied to eliminate outliers, so that we could evaluate the distribution and variability of the data.

Prior to miRNA analysis, the raw data underwent processing with Bcl2fastq software (v 2.20). This program eliminated sequences that had poly-N, had 5’ adapters, did not have 3’ adapters or insert fragments, and had an excessive amount of poly-A, T, G, or C. We computed quality control measures for the clean data, which include Q20, Q30, and GC content. Following the selection of sequences that were within a certain length range, Bowtie software (v1.3.1) was used to align them to the reference genome (20). The miRBase database was used to accomplish miRNA annotation. Just like with the mRNA analysis, we used expectation maximization to determine the levels of gene and transcript expression, and FK-MMR to normalize the expression data. For further quality control, box and PC plots were created using the ggplot2 software (v 3.3.6).

### 2.6 Differentially expressed analysis and reverse intersection

Using the DESeq2 program (v 1.38.0) (21) on the preprocessed transcriptome data, we conducted differential expression analysis comparing the KO vs. control (CO) and OE vs. CO groups to assess the effects of PDCD4 deletion and overexpression on gene, LNC-RNA, and miRNA expression in mice. Separately, the entities that showed differential expression were called DEGs, differentially expressed long non-coding RNAs (DE-LncRNAs), and differentially expressed microRNAs (DE-miRNAs). For this study, the significance level was established at |log2 fold change (FC)| > 0.5 and adjusted p-value (adj.p) < 0.05. To illustrate the expression patterns, heatmaps were made using the ggplot2 package (v 3.3.6) and volcano plots were created using the pheatmap package (v 1.0.12) (22). Top 10 genes that were upregulated and downregulated based on log2FC values were highlighted. By utilizing the ggVenn package (v1.2.2) (23), we were able to do reverse intersection analysis and discover shared differentially expressed components across categories. By combining the common DEGs from the KO vs. CO and OE vs. CO comparisons, this method produced intersection DEGs. Also produced using the same procedure were intersection DE-miRNAs and intersection DE-LncRNAs.

### 2.7 Enrichment analysis

The clusterProfiler package (v 4.7.1.3) (24) was used with a significance threshold of p < 0.05 to conduct enrichment analyses in Gene Ontology (GO) and the Kyoto Encyclopedia of Genes and Genomes (KEGG), in order to explore the possible biological functions and pathways linked to the intersection of differentially expressed mRNAs. An ascending gene ratio was used to arrange the GO and KEGG analysis findings. The three most abundant keywords and the 10 most important gene functions were shown independently for each group. With this method, we were able to zero down on the specific biological mechanisms and molecular pathways that may play a role in the control of genes related to PDCD4 in patients with atrial fibrillation.

### 2.8 Construction of protein-protein interaction network

After that, we looked for protein-level interactions between these genes by entering the intersection of differentially expressed mRNAs into the STRINGP database with a confidence score threshold of > 0.4. After removing the genes that weren’t part of the network, the remaining genes were thought of as important interacting proteins. Cytoscape software (v3.8.1) (PMID: 31477170) was used to display the resultant PPI network, which allowed for a thorough depiction of the molecular interactions within the atrial fibrillation PDCD4-associated gene network.

### 2.9 Construction of relevant molecular regulatory networks

The “cor” function in the psych package (v2.4.3) was used to do a Spearman correlation study between mRNAs and differentially expressed LncRNAs (PMID: 37505622). The pheatmap software (v1.0.12) was used to create the heatmaps, using the criteria of |correlation| > 0.9 and p < 0.05. The key genes were found by combining these findings with miRNA predictions for the mRNAs that showed differential expression, which were obtained from the miRanda and PITA databases. The ChEA3 database was searched for predicted TFs associated with these important genes, using a score cutoff greater than 900. To better understand the molecular interactions that lead to atrial fibrillation, a regulatory network involving microRNAs (miRNAs) and transferases (TFs) was built and shown in Cytoscape (v3.8.1).

### 2.10 Key genes localization

We used the RCircos software (v1.2.2) (PMID: 23937229) to look into the chromosomal locations of the important genes according to the gene annotation data. In order to make the chromosomal data visualizable, we first isolated the positions of the important genes. The genomic distribution of the important genes was then clearly shown by creating circumcos plots, which show where the genes are located on the chromosomes.

### 2.11 Enrichment analysis of key genes

We used the clusterProfiler software (v4.7.1.3) to do GO and KEGG analyses, and we show you the top three GO keywords and top ten KEGG pathways. The psych package (v2.4.3) was used to determine the Spearman correlation coefficients between all of the genes and each important gene. Applying the GSEA program to the transcriptome dataset allowed us to detect route differences between the KO vs. CO and OE vs. CO groups. The expression of several pathways was examined using DESeq2 (v1.38.0), and pathways were considered significant if their adjusted p-value was less than 0.05. For the purpose of visualizing the results, bar plots were created using ggplot2 (v3.3.6). In order to do pathway analysis, the Molecular Signatures Database’s (MOSD) “m2.all.v2023.2.Mm.symbols.gmt” gene set was borrowed.

After sorting the gene correlation coefficients by decreasing order, the clusterProfiler software (v4.10.1) and the same MOSD reference gene set were used to conduct Gene Set Enrichment Analysis (GESEAN). The five routes that were shown to be considerably enhanced (adj. p < 0.05) were then displayed. After sorting the gene correlation coefficients by decreasing order, the clusterProfiler software (v4.10.1) and the same MOSD reference gene set were used to conduct Gene Set Enrichment Analysis (GESEAN). The five routes that were shown to be considerably enhanced (adj. p < 0.05) were then displayed.

### 2.12 Drug prediction and molecular docking

By merging drug data from the Drug Gene Interaction Database (DUGID) and the Comparative Toxicogenomics Database (COTD), with an emphasis on interactions with an Interaction Count > 1, candidate compounds were chosen to find possible therapeutic medicines targeting the important genes. Using Cytoscape (v3.8.1), we were able to see how these potential drugs interacted with important genes. In order to assess the binding affinity of important genes with the most potential medications, the Protein Data Bank (PDB) was queried for the protein structures of the relevant genes and the PubChem database was queried for two-dimensional molecular structures of the chosen compounds. To conduct molecular docking investigations, we used AutoDock Vina after converting protein structures to PDBQT format with AutoDock software. A binding energy criterion of less than -5 kcal/mol was used to show the docking data in PyMOL (v3.0) (PMID: 31710740).

### 2.13 Disease association analysis

Using the COTD an association analysis between the key genes and various diseases was performed. After the results were sorted based on the Inference Score, the top 20 diseases were visualized using Cytoscape software (v 3.8.1).

### 2.14 Identification of differential cells based on SCRNA-SEQ data

The CreateSeuratObject function from the Seurat package (v4.3.0) (PMID: 34062119) was used to preprocess the GSE197518 dataset. The settings were set to min.cells = 3 and min.features = 200. We used the PercentageFeatureSet tool to determine the content of mitochondrial genes; cells whose mitochondrial gene expression was more than 10% were not included. Any cells or genes that did not match the quality control standards, which included minGene = 200, maxGene = 6000, and pctMT < 10, were eliminated. To see the quality control results graphically, we used the ggplot2 software (v3.3.6).

After using NormalizeData to standardize the data, the FindVariableFeatures function used the mean-variance relationship to identify the top 2000 genes with the highest levels of variability. For the top ten most variable genes, these genes were shown visually with labelled annotations. Following data normalization with ScaleData, dimensionality reduction was achieved by doing principal component (PC) analysis on the variable genes using the runPC function. To find the best number of PCs to cluster, the ElbowPlot program looked for ones that were before the elbow point.

The FindClusters and FindNeighbors functions were used to do unsupervised clustering at a resolution of 0.4. Optimizing cluster assignments was done using a Jackstraw-based permutation test, which helped to refine clustering. Using the clustering visualization tools provided by Seurat, the resulting clusters were shown.

Marker gene expression was used to identify cell types for each cluster, with common markers and established markers from the literature (PMID: 37440641) consulted. Using ggplot2 (v3.3.6), a dot plot was created to confirm the marker gene expression across clusters.

To reduce potential technical confounders in cell type proportion comparisons, stringent quality control filtering was applied, and data normalization steps were conducted to mitigate batch effects and sequencing depth variability. The Jackstraw permutation test further ensured robust clustering results, minimizing technical artifact influence on biological interpretation.

### 2.15 Identification of key cells and gene expression analysis

Stack bar plots were created using the ggplot2 software (v3.3.6) to portray the proportions of each cell type inside clusters. We used box plots to show the levels of expression of important biomarkers in immune cells. The Wilcoxon rank-sum test was used to identify cells with significantly different gene expression, and these cells were defined as important cells, with an adjusted p-value threshold of less than 0.05. In the end, we utilized the FeaturePlot function from the Seurat package (v4.3.0) to generate UMAP plots, which displayed the gene expression patterns in the selected key cells.

### 2.16 Intercellular communication analysis

Using the CellChat package (v 1.6.1) (PMID: 33597522), intercellular communication analysis was performed on all annotated cell clusters. The results were then visualized as interaction plots alongside a heatmap to depict the communication patterns between the different cell types.

### 2.17 Gene expression and mutation analysis in pan-cancer datasets

We obtained RNA sequencing data (FPKM format) and somatic mutation profiles for 33 cancer types from The Cancer Genome Atlas (TCGA) via the UCSC Xena browser. PDCD4 expression was compared between tumor and adjacent normal tissues using the Wilcoxon rank-sum test. The tumor mutational burden (TMB) and microsatellite instability (MSI) scores were acquired from published pan-cancer annotations. Spearman correlation was used to evaluate the association between PDCD4 expression and TMB/MSI in each cancer type.

### 2.18 Survival analysis across cancers

Overall survival (OS) data were retrieved from TCGA clinical annotations. Kaplan–Meier survival curves were plotted using the “survival” and “survminer” R packages to assess the prognostic significance of PDCD4 expression. Patients were stratified into high and low expression groups based on the median PDCD4 level within each cancer type. The log-rank test was applied to assess significance. Multivariate Cox regression was not performed in this study, which we acknowledge as a limitation.

### 2.19 Immune infiltration analysis

To explore the immune relevance of PDCD4, we assessed its correlation with immune cell infiltration using two independent approaches: CIBERSORT and single-sample gene set enrichment analysis (ssGSEA). The LM22 gene signature matrix was used in CIBERSORT to estimate the relative proportions of 22 immune cell types. ssGSEA was performed using the “GSVA” R package and immune-related gene sets from published literature. Spearman correlation was used to evaluate associations between PDCD4 expression and infiltration scores.

### 2.20 Statistical analysis

R program (v4.2.2) was used to perform bioinformatics analysis. When comparing groups in animal studies, the Wilcoxon rank-sum test was used because the data was non-parametric. If the adjusted p-value (adj.p) was less than 0.05, it was deemed statistically significant. All analyses were corrected for multiple testing, and the ggplot2 package (v3.3.6) was used to construct visualizations of the results.

## 3 Results

### 3.1 Differentially expressed genes related to PDCD4 and their functional analysis

**Figure 1:**
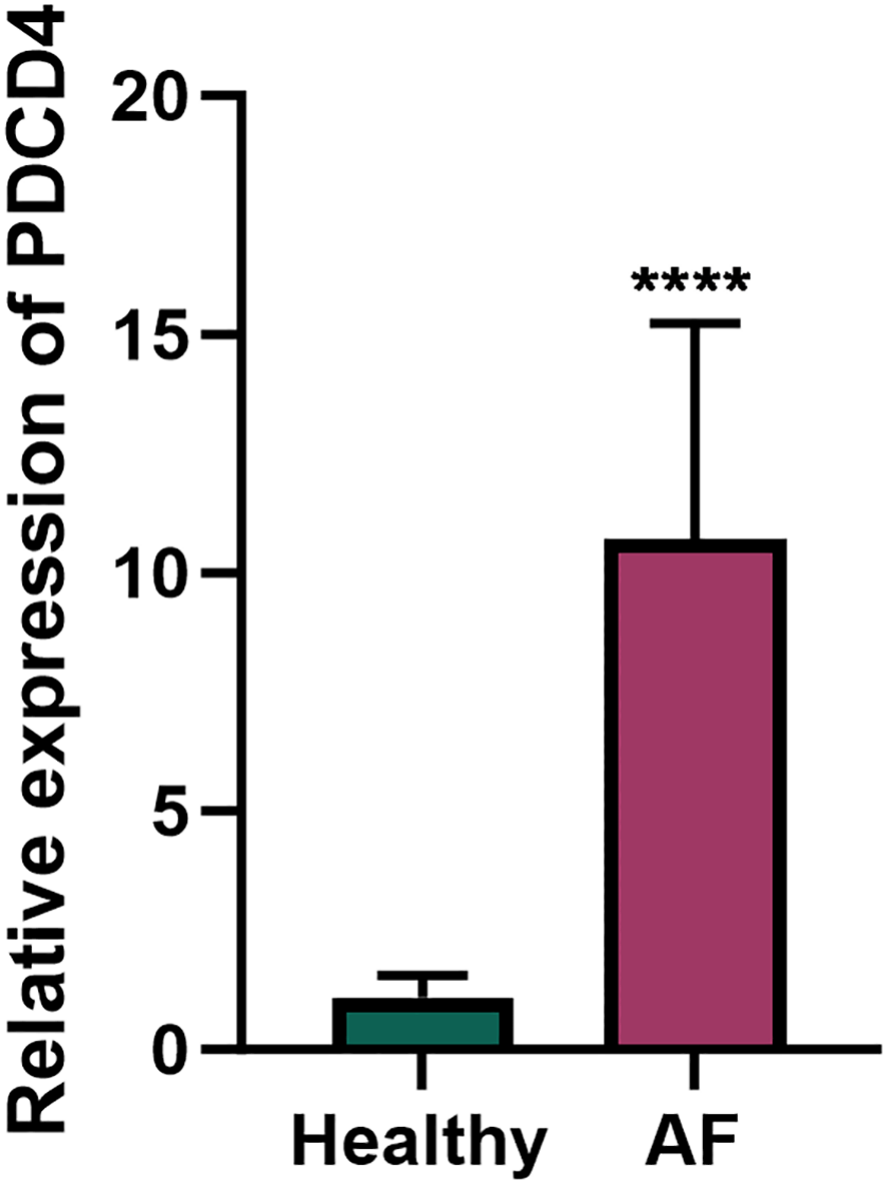
PDCD4 expression in peripheral blood mononuclear cells (PBMCs) of atrial fibrillation (AF) patients and healthy controls. Bar chart showing the relative expression levels of PDCD4 in PBMCs from AF patients (n=5) and healthy controls (n=5), determined by qRT-PCR; "****" indicates p < 0.0001. GAPDH was used as the internal control. Statistical significance was assessed using an unpaired t-test.

**Figure 2:**
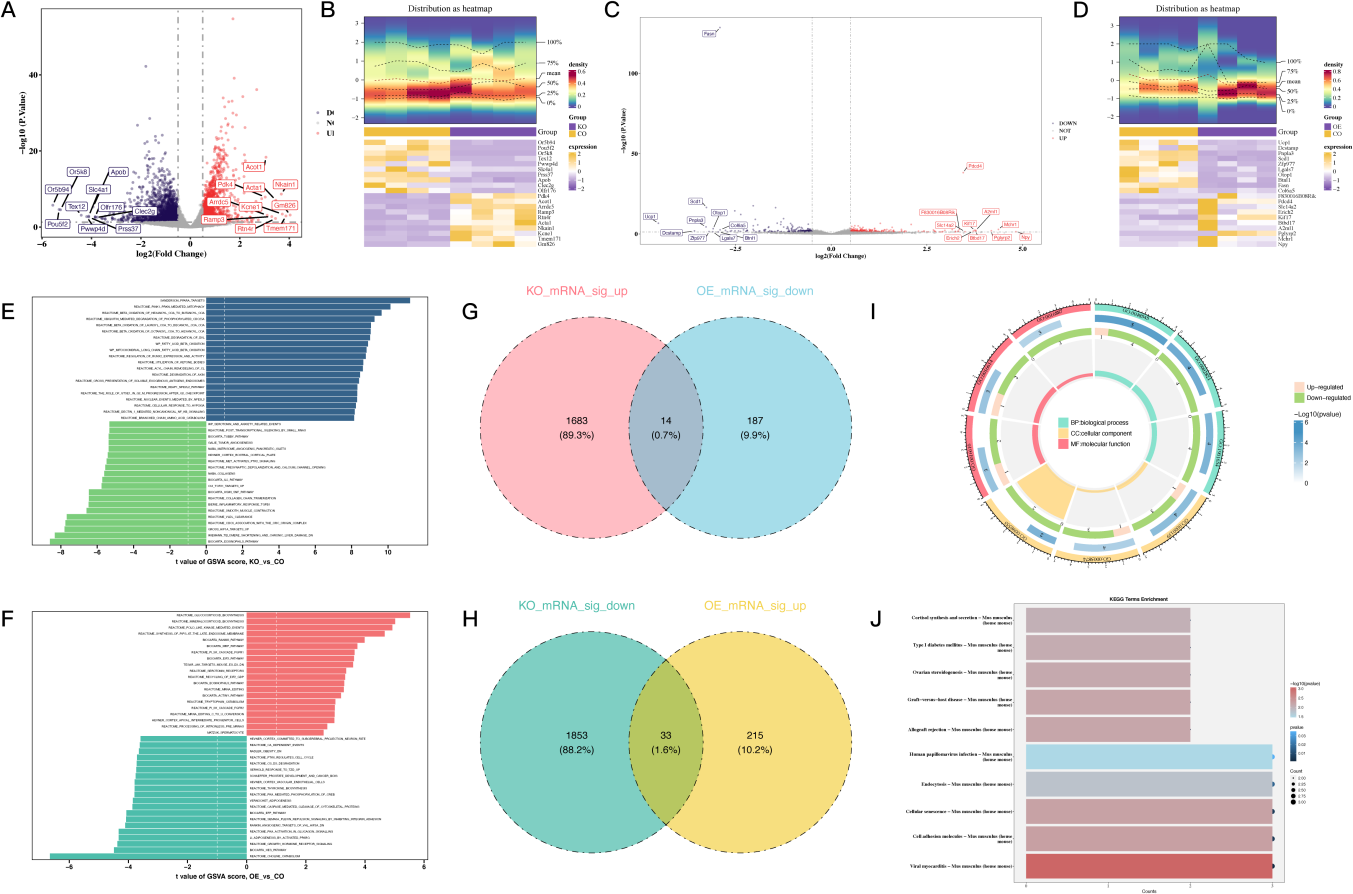
Differential gene expression and functional analysis associated with PDCD4 regulation in atrial fibrillation. **(A)** Volcano plot illustrating DEGs in the PDCD4-KO group compared to the control group, highlighting the top 10 upregulated and downregulated genes. **(B)** Heatmap showing the expression levels of the top 10 upregulated and downregulated DEGs in the PDCD4-KO group compared to the control group. **(C)** Volcano plot illustrating DEGs in the PDCD4-OE group compared to the control group, highlighting the top 10 upregulated and downregulated genes. **(D)** Heatmap showing the expression levels of the top 10 upregulated and downregulated DEGs in the PDCD4-OE group compared to the control group. **(E)** Bar chart of GSEA results for pathway enrichment analysis in the PDCD4-KO group. **(F)** Bar chart of GSEA results for pathway enrichment analysis in the PDCD4-OE group. **(G)** Venn diagram showing overlapping genes significantly upregulated in the PDCD4-KO group and downregulated in the PDCD4-OE group. **(H)** Venn diagram showing overlapping genes significantly downregulated in the PDCD4-KO group and upregulated in the PDCD4-OE group. **(I)** Circular plot summarizing GO enrichment analysis for intersecting DEGs. **(J)** Bar chart summarizing KEGG enrichment analysis for intersecting DEGs.

To investigate the role of PDCD4 in AF, we first examined its expression levels in PBMCs from AF patients and healthy controls. qRT-PCR analysis revealed a significant upregulation of PDCD4 in the PBMCs of AF patients compared to controls (**Figure 1**), suggesting a potential role for PDCD4 in the pathogenesis of AF. To further elucidate the regulatory mechanisms and functional significance of PDCD4 in AF, we conducted transcriptome sequencing. After rigorous quality control, the sequencing data showed minimal variation across samples and consistent fragment size distribution (**Supplementary Figure 1**). Principal component (PC) analysis confirmed clear separation between groups, indicating reliable sample classification (**Supplementary Figure 1**). Differential expression analysis identified a total of 3,583 differentially expressed genes (DEGs) in the PDCD4 knockout (PDCD4-KO) group compared to controls, with 1,697 genes upregulated and 1,886 genes downregulated (**Figures 2A, B**). Similarly, 449 DEGs were detected in the PDCD4 overexpression (PDCD4-OE) group, with 248 upregulated and 201 downregulated genes (**Figures 2C, D**). To explore the biological pathways associated with PDCD4, we performed gene set enrichment analysis (GSEA). In the PDCD4-KO group, GSEA revealed reduced eosinophil-related signatures and enhanced beta-oxidation of hexanoyl-CoA to butanoyl-CoA (**Figure 2E**). Conversely, the PDCD4-OE group exhibited downregulated choline catabolism and upregulated glucocorticoid biosynthesis (**Figure 2F**). To identify key regulatory genes, we intersected the DEGs from both comparisons and identified 47 common differentially expressed mRNAs (DE-mRNAs) as candidates for further analysis (**Figures 2G, H**). Gene Ontology (GO) enrichment analysis of these DE-mRNAs revealed significant associations with biological processes such as steroid hormone response, positive regulation of adaptive immune responses, and response to ketone. As shown in **Figure 2I**, cellular component analysis highlighted enrichment in the Golgi membrane, microtubules, and the lumenal side of the endoplasmic reticulum membrane. In terms of molecular function, the predominant roles involved oxidoreductase activity, particularly those involving NAD(P)+ as acceptors, as well as ATP hydrolysis activity. Finally, KEGG pathway enrichment analysis (**Figure 2J**) identified significant pathways related to viral myocarditis, cell adhesion molecules, and cellular senescence. Taken together, these findings shed light on the multifaceted biochemical and molecular pathways associated with PDCD4 and underscore its potential role in the development of atrial fibrillation.

### 3.2 The PPI network and the functional findings of eleven important genes related to PDCD4

**Figure 3:**
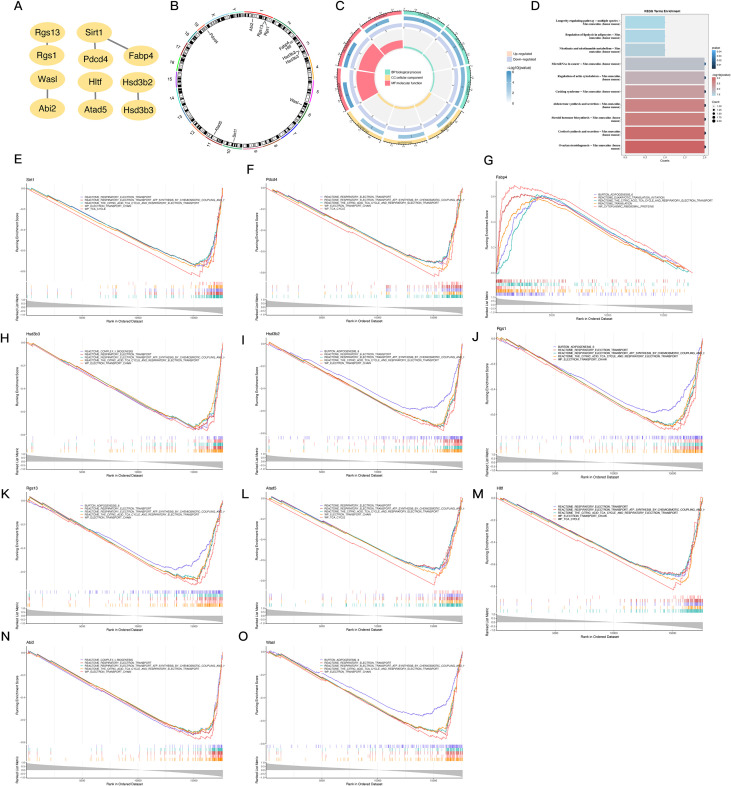
PPI network construction, chromosomal localization, and functional enrichment analysis of 11 PDCD4-associated key genes. **(A)** PPI network constructed using STRINGP for 47 candidate genes, with 11 nodes and 6 edges retained after removing isolated nodes. **(B)** Chromosomal localization of the 11 key genes visualized using RCircos, displaying their distribution across specific chromosomes. **(C)** Circular plot summarizing GO enrichment analysis for the 11 key genes, categorized by biological processes, cellular components, and molecular functions. **(D)** Bar chart summarizing KEGG pathway enrichment analysis for the 11 key genes. **(E–O)** GESEAN results for the 11 key genes, showing enrichment in distinct pathways related to energy metabolism, mitochondrial function, and specialized metabolic processes.

Our goal in building the PPI network was to gain a better understanding of the protein-level interactions among the 47 DE-mRNAs. **Figure 3A** shows that after isolating nodes, 11 proteins with 6 different kinds of interactions remained and were subsequently recognized as important genes. A targeted subset of prospective genes for further investigation is represented by these important genes. Based on the results of the chromosomal localization study, it was found that the following genes did not distribute randomly: Abi2, Rgs13, and Rgs1 were grouped on chromosome 1; Fabp4, Hltf, Hsd3b2, and Hsd3b3 were on chromosome 3; Wasl was on chromosome 6; Sirt1 was on chromosome 10; Atad5 was on chromosome 11; and PDCD4 was on chromosome 19 in **Figure 3B**. By using GO enrichment analysis, we were able to probe the discovered important genes’ biological importance. The primary associations of the important genes in the biological process category with steroid biosynthesis pathways, ketones, and steroid hormones indicate that these genes are involved in metabolic and endocrine control. This group of cellular components includes genes that are involved in mitochondrial function and intracellular structural organization; these genes were shown to be more abundant in the intercellular bridge, organelle envelope lumen, and mitochondrial intermembrane space. Their enzymatic activities in steroid metabolism were reflected in the genes’ molecular function links to 3-beta-hydroxy-delta5-steroid dehydrogenase, intramolecular oxidoreductase, and steroid delta-isomerase (**Figure 3C**). **Figure 3D** shows the results of further KEGG pathway analysis that connected these genes to important metabolic and regulatory processes, such as steroid hormone biosynthesis, cortisol production and release, and ovarian steroidogenesis. Additional insights were provided by GESEAN, which showed that pathways related to respiratory electron transport, ATP synthesis by chemiosmotic coupling, and heat production by uncoupling proteins were significantly enriched in 10 out of the 11 key genes (Sirt1, PDCD4, Hsd3b3, Hsd3b2, Rgs1, Rgs13, Atad5, Hltf, Abi2, and Wasl). This emphasizes the functions of the mitochondria and their significance in energy metabolism. **Figures 3E–O** show that Fabp4 has a specific role in metabolism, since it is uniquely enriched in the citric acid (TCA) cycle. To summarize, the results of the enrichment analyses strongly suggest that these important genes are involved in hormone pathways, steroid metabolism, and mitochondrial energy generation. This suggests that they may play a role in atrial fibrillation and the metabolic dysregulation that comes with it.

### 3.3 Building regulatory networks for PDCD4-related key genes using LNC-RNA, miRNA, and TFs

**Figure 4:**
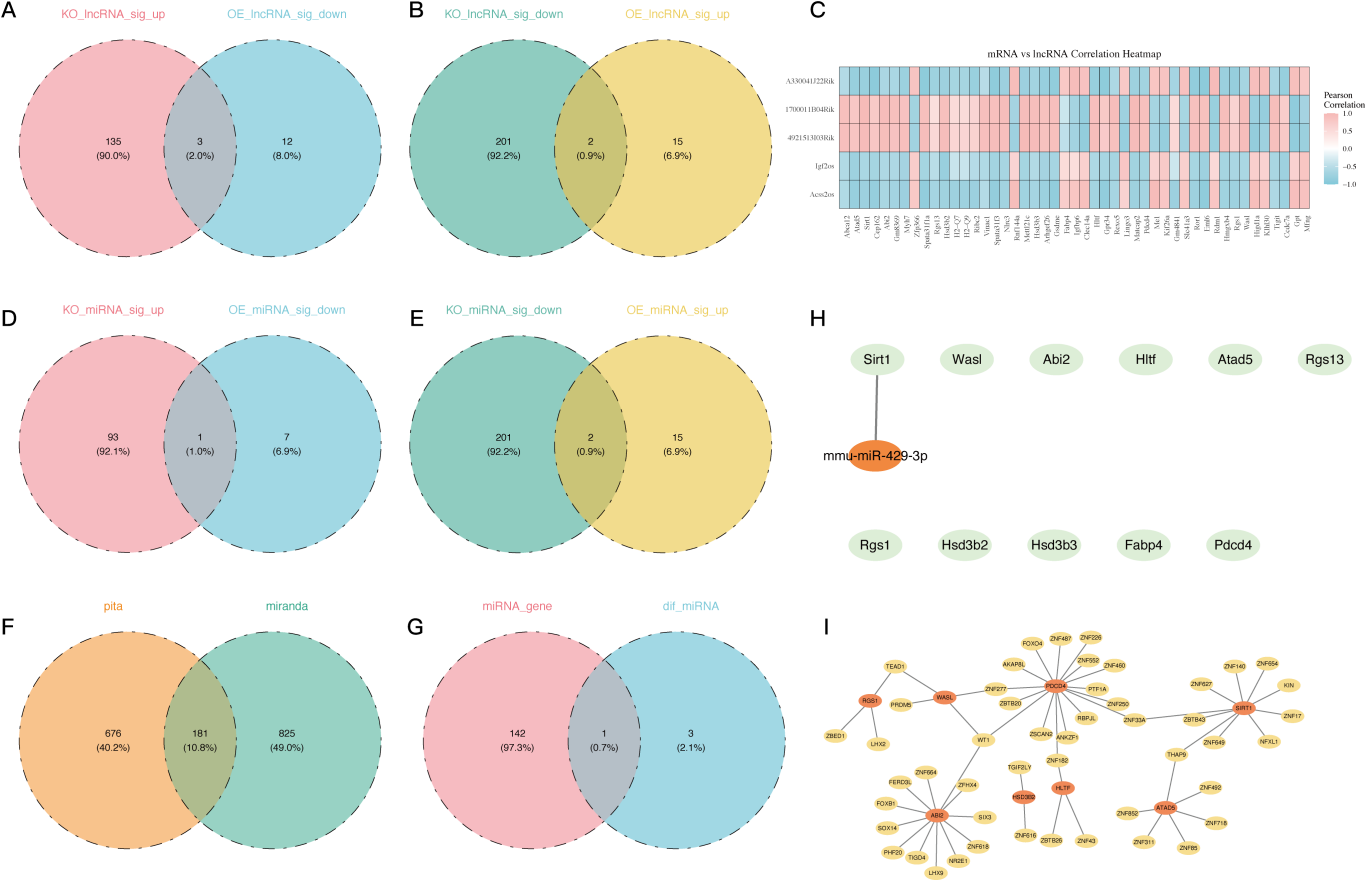
Regulatory network analysis of LncRNAs, miRNAs, and TFs associated with 11 PDCD4-related key genes. **(A)** Venn diagram showing overlapping LNC-RNA significantly upregulated in the PDCD4-KO group and downregulated in the PDCD4-OE group. **(B)** Venn diagram showing overlapping LNC-RNA significantly downregulated in the PDCD4-KO group and upregulated in the PDCD4-OE group. **(C)** Heatmap illustrating the correlation between 5 intersecting differentially expressed LncRNAs and the 11 key genes. **(D)** Venn diagram showing overlapping miRNA significantly upregulated in the PDCD4-KO group and downregulated in the PDCD4-OE group. **(E)** Venn diagram showing overlapping miRNA significantly downregulated in the PDCD4-KO group and upregulated in the PDCD4-OE group. **(F)** Venn diagram showing the overlap of miRNAs targeting differentially expressed genes as predicted by PITA and miRanda databases, with 181 common miRNAs identified. **(G)** Venn diagram showing the intersection of database-predicted miRNAs targeting PDCD4-associated genes and miRNAs differentially expressed in sequencing data. **(H)** Key miRNA-mRNA regulatory network visualized using Cytoscape **(I)** Transcription factor regulatory network of the 11 PDCD4-related key genes.

Next, the LncRNAs were annotated using the TANRIC and LncRBase databases, after the alignment of the clean reads to the reference genome. Density and boxplot analyses of the expression distributions revealed comparable expression levels among samples (**Supplementary Figure 2A**). Additional assurance of data reliability was provided by PC analysis, which aimed to detect and eliminate outliers (**Supplementary Figure 2B**). By contrasting the PDCD4-KO and control groups, in addition to the PDCD4-OE and control groups, a differential expression analysis was conducted. In the PDCD4-KO vs. control comparison, 341 DEGs were shown by volcano plots and heatmaps (**Supplementary Figures 2C-F**). Of these, 138 genes were upregulated and 203 genes were downregulated. In the PDCD4-OE vs. control comparison, 32 genes were shown, with 17 genes upregulated and 15 genes downregulated. By combining the DEGs from the two sets of comparisons, five possible LncRNAs were found (**Figures 4A, B**). A correlation study was carried out to investigate the co-expression connection between the DE-LncRNAs and DE-mRNAs. **Figure 4C** shows that there is a high association between the DE-mRNAs and the LncRNAs 4921513I3Rik and 1700011B04Rik.

Following the processing and annotation of miRNA sequencing data (**Supplementary Figures 2G-H**), differential miRNA analysis was conducted across the groups (**Supplementary Figures 2I-L**) to study the regulatory mechanisms of DE-miRNAs on the eleven DE-mRNAs that had been discovered. The intersection of the differential miRNAs led to the identification of three potential DE-miRNAs (**Figures 4D, E**). We were able to identify 181 target miRNAs by merging the PITA and miRanda datasets (**Figure 4F**). A crucial miRNA that controls Sirt1 was identified as mmu-miR-429-3p by further intersecting with the differentially expressed miRNAs in our analysis (**Figures 4G, H**).

In addition, a TF-mRNA network was built using 11 important genes and 49 projected TFs as there were no predicted TFs for the crucial genes RGS13, FABP4, and HSD3B3. **Figure 3I** shows that Wt1 typically regulates PDCD4, Wasl, and Abi2, whereas Thap9 typically regulates Sirt1 and Atad5.

### 3.4 In the heart microenvironment of atrial fibrillation, characterization of critical gene expression and cellular interactions

**Figure 5:**
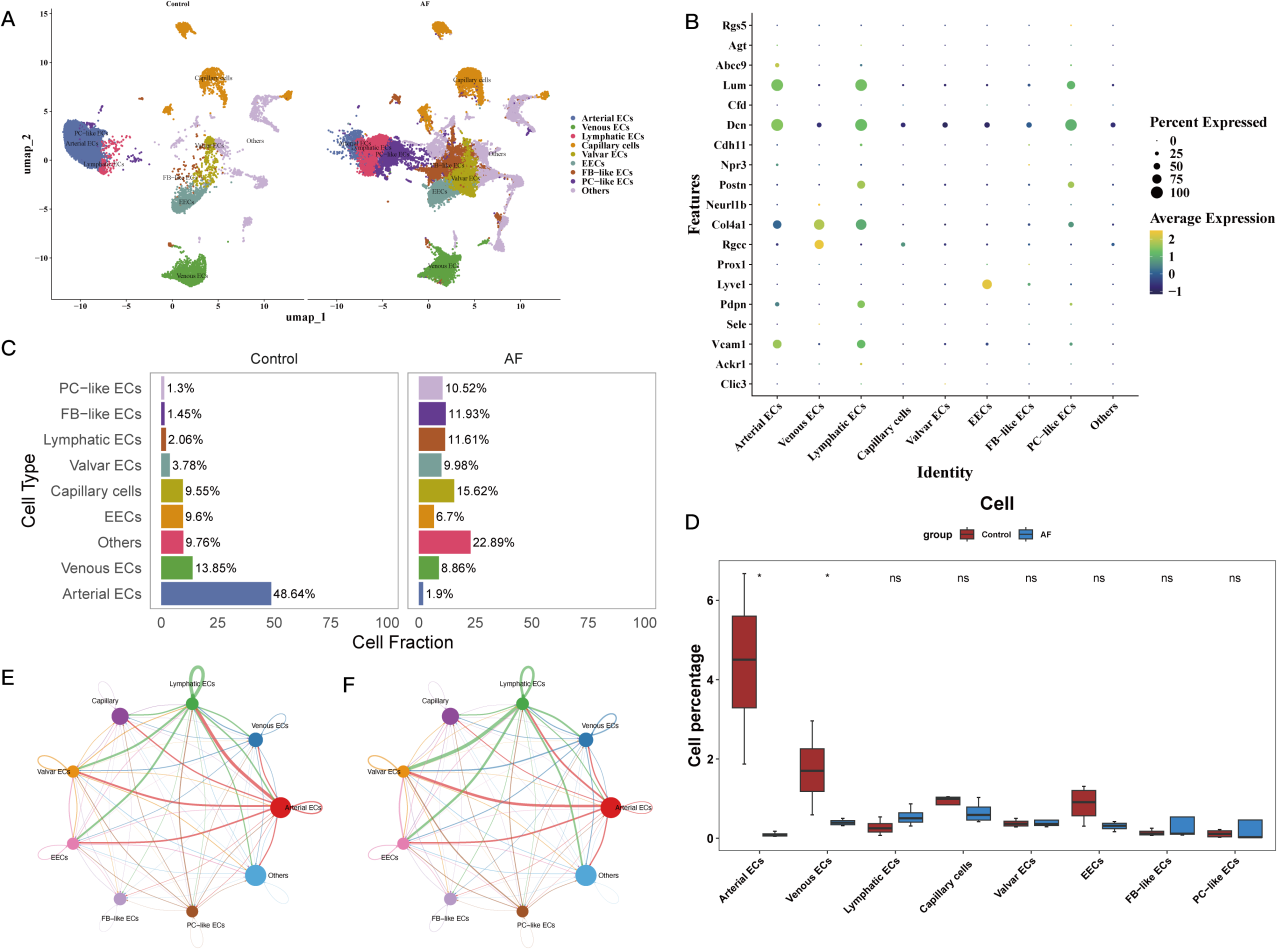
Characterization of cell type composition and interactions in the cardiac microenvironment of atrial fibrillation. **(A)** UMAP plot showing eight major cell subtypes identified via SCRNA-SEQ, including Arterial ECs, Venous ECs, Lymphatic ECs, Capillary ECs, Valvular ECs, EECs, FB-like ECs, and PC-like ECs. **(B)** Bubble plot illustrating the expression of marker genes specific to each identified cell subtype. **(C)** Bar chart displaying the cell type composition in the control and AF groups. **(D)** Comparative analysis of cell type proportions between the AF and control groups. **(E, F)** Cell-cell communication analysis between eight cell subtypes.

Preprocessing the public single-cell RNA sequencing dataset GSE197518 allowed us to examine the cardiac microenvironment and the function of important genes in various cell types (**Supplementary Figures 3A-E**). **Supplementary Figures 3F, G** show the identification of 21 separate cell clusters following quality inspection. **Figures 5A, B** shows that these clusters were categorized as seven distinct cell types and one unidentified subgroup according to gene expression patterns and references in the literature. After that, we looked at the different cell types in both the AF and control groups. The AF group had a preponderance of capillary endothelial cells (ECs), in contrast to the control group which exhibited a preponderance of arterial ECs (**Figure 5C**). In **Figure 5D**, we can see that the AF group had substantially lower arterial and venous ECs. We used the “CellChat” program to analyze cell-cell communication in order to learn more about cellular interactions in the heart microenvironment. According to the results of this investigation, lymphatic ECs and artery ECs interact strongly (**Supplementary Figures 4E, F**; **Figures 5E, F**). Lastly, we looked at the expression of eleven important genes in various cell types. Our findings demonstrated that venous ECs were the primary locus of Rgs1 expression, whereas arterial ECs were the primary locus of Fabp4 and Wasl expression. The AF group exhibited a preponderance of PDCD4-expressing capillary cells and Sirt1-expressing fibroblast-like endothelial cells, respectively. The expression of Hsd3b2, Rgs13, and Atad5 was found solely in AF cells, although at extremely low levels; in contrast, Abi2 and Hltf were mostly expressed in venous ECs (**Supplementary Figures 4A-J**).

### 3.5 Cancer risk factor analysis, molecular docking, and targeted drug prediction for important genes

**Figure 6:**
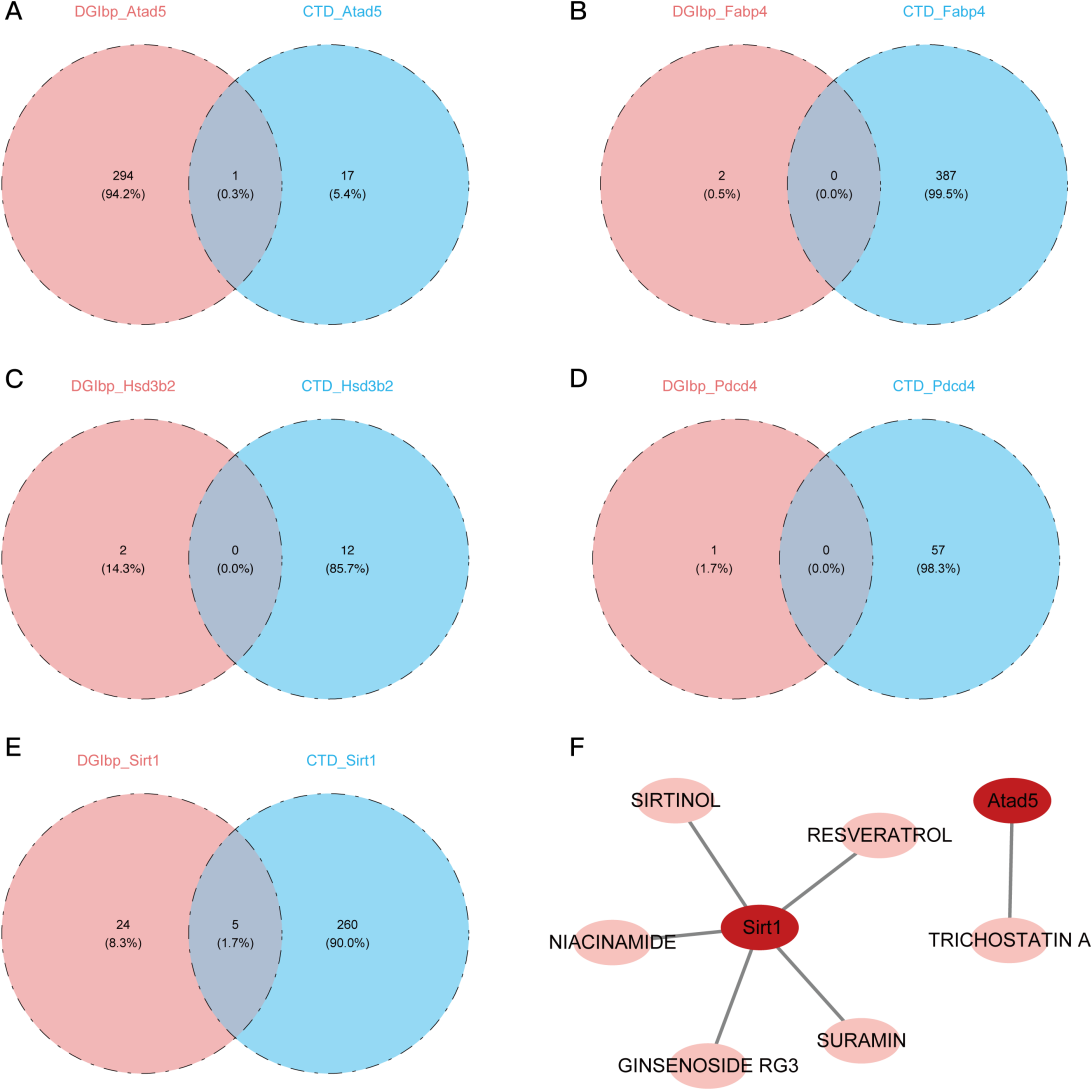
Prediction and identification of potential drugs targeting key genes. **(A)** Venn diagram showing the intersection of potential drugs targeting ATAD5 predicted by DUGID and COTD databases. **(B)** Venn diagram showing the intersection of potential drugs targeting FABP4 predicted by DUGID and COTD databases. **(C)** Venn diagram showing the intersection of potential drugs targeting HSD3B2 predicted by DUGID and COTD databases. **(D)** Venn diagram showing the intersection of potential drugs targeting PDCD4 predicted by DUGID and COTD databases. **(E)** Venn diagram showing the intersection of potential drugs targeting SIRT1 predicted by DUGID and COTD databases. **(F)** Identification of specific drugs targeting SIRT1, including SURAMIN, SIRTINOL, RESVERATROL, NIACINAMIDE, and GINSENOSIDE RG3, as well as a drug targeting ATAD5, TRICHOSTATIN A.

Out of the five genes we tested, 295 were associated with Atad5, 2 with Fabp4, 2 with Hsd3b2, 1 with PDCD4, and 29 with Sirt1. And results show that a separate study utilizing the COTD database predicted the effects of 18 medications on Atad5, 387 on Fabp4, 12 on Hsd3b2, 57 on PDCD4, and 265 on Sirt1. One medication candidate for Atad5 (sirtinol) and five for Sirt1 (trichostatin A) were chosen for further molecular docking investigations when the drug predictions from both databases intersected (**Figures 6A–F**).

**Figure 7:**
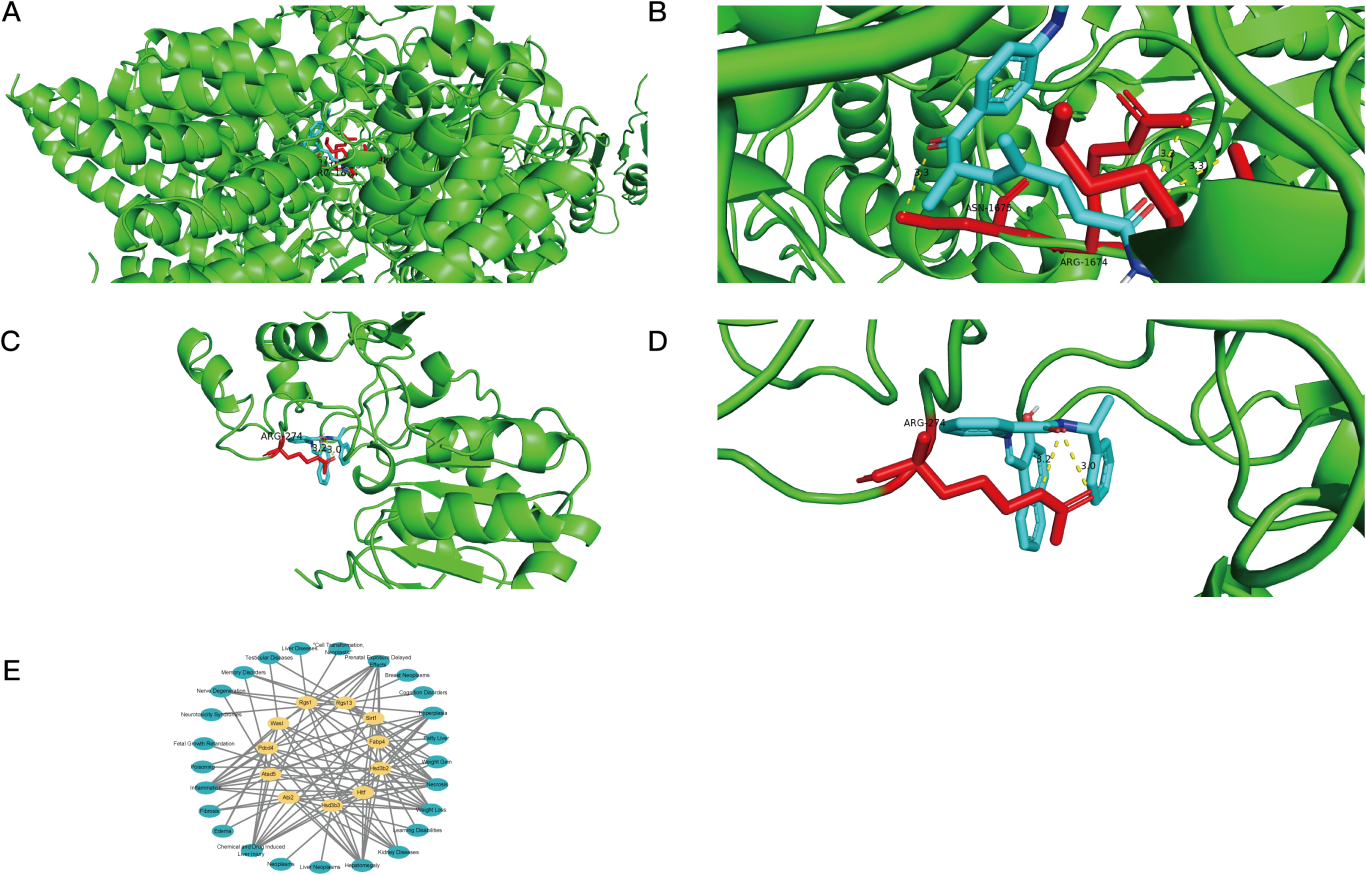
Molecular docking and disease associations of key genes. **(A, B)** Visualization of molecular docking results showing the interaction between ATAD5 and Trichostatin A. Blue helices represent the protein molecule, yellow dashed lines indicate hydrogen bonds, colored ring structures represent the drug ligand, and red bars mark the protein binding sites of the drug ligand. **(C, D)** Visualization of molecular docking results showing the interaction between SIRT1 and Sirtinol. Blue helices represent the protein molecule, yellow dashed lines indicate hydrogen bonds, colored ring structures represent the drug ligand, and red bars mark the protein binding sites of the drug ligand. **(E)** Analysis showing the relationships between key genes and various diseases.

Molecular docking analysis showed that the target genes and their anticipated pharmaceuticals had high binding affinities. Both Sirt1 and Atad5 demonstrated strong binding performance with trichostatin A and sirtinol, respectively, with docking scores of -8.5 and -7.9 kcal/mol, respectively, suggesting binding energies below -5 kcal/mol (**Figures 7A–D**).

The disease-related network further showed that the 11 critical genes were strongly connected with inflammation and chemical-or drug-induced liver damage (**Figure 7E**). The results shed light on possible treatments and the importance of the important genes in illness settings, providing important information for the development of new treatments.

### 3.6 PDCD4 expression across cancer types and its prognostic significance

**Figure 8:**
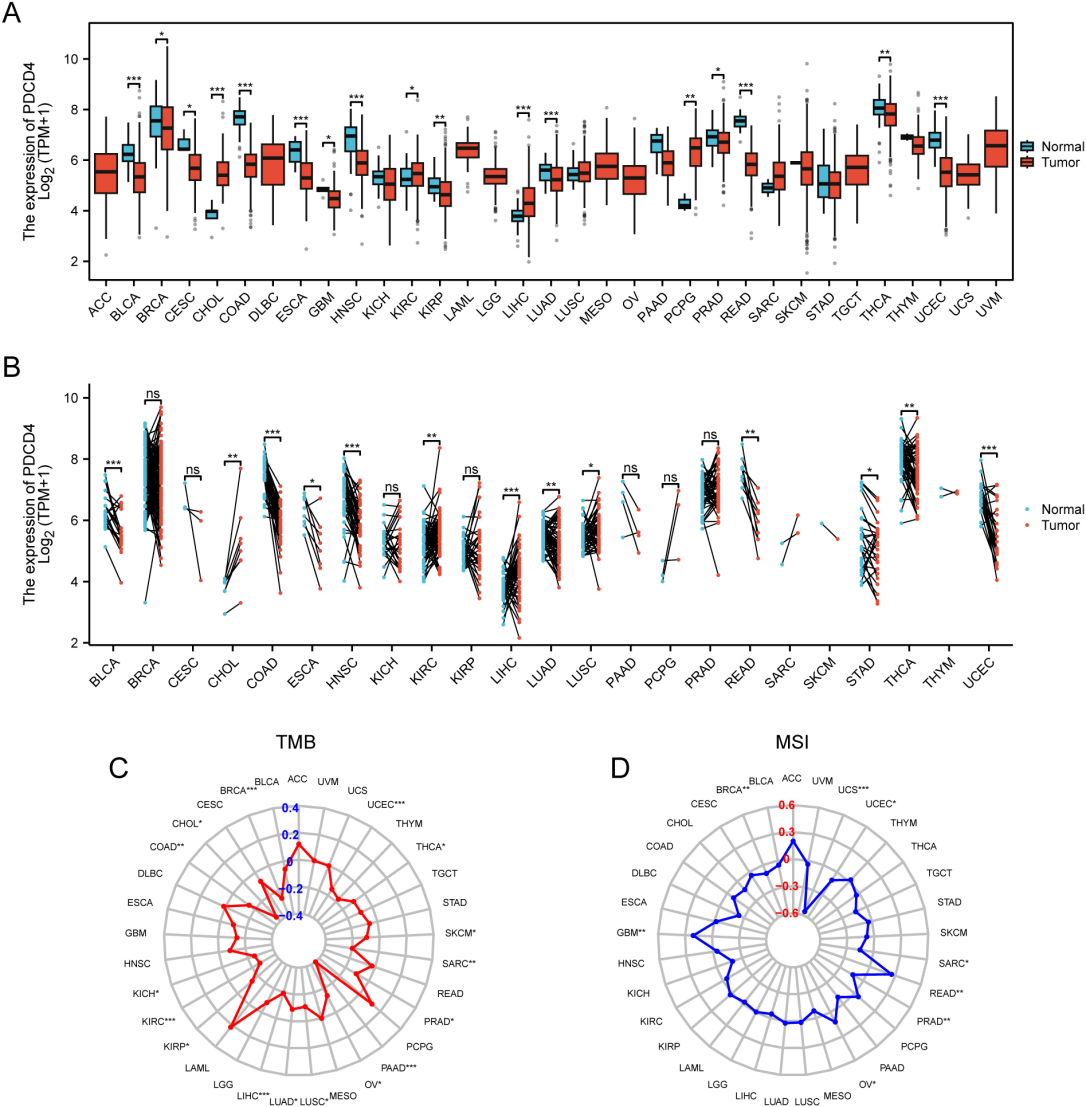
Pan-cancer analysis of PDCD4 expression and mutational characteristics. **(A)** Boxplot showing PDCD4 expression levels across different cancer types. Tumor tissues (red) and normal tissues (blue) are compared to highlight expression differences. **(B)** Paired sample analysis illustrating changes in PDCD4 expression between tumor and matched normal tissues from the same patients, with black lines connecting paired samples. **(C)** Radar plot depicting the correlation between PDCD4 expression and TMB across various cancer types, with significance levels indicated. **(D)** Radar plot showing the correlation between PDCD4 expression and MSI in different cancers, with significance levels indicated. Statistical significance is marked as follows: *p < 0.05; **p < 0.01; ***p < 0.001. ns, not significant.

**Figure 9:**
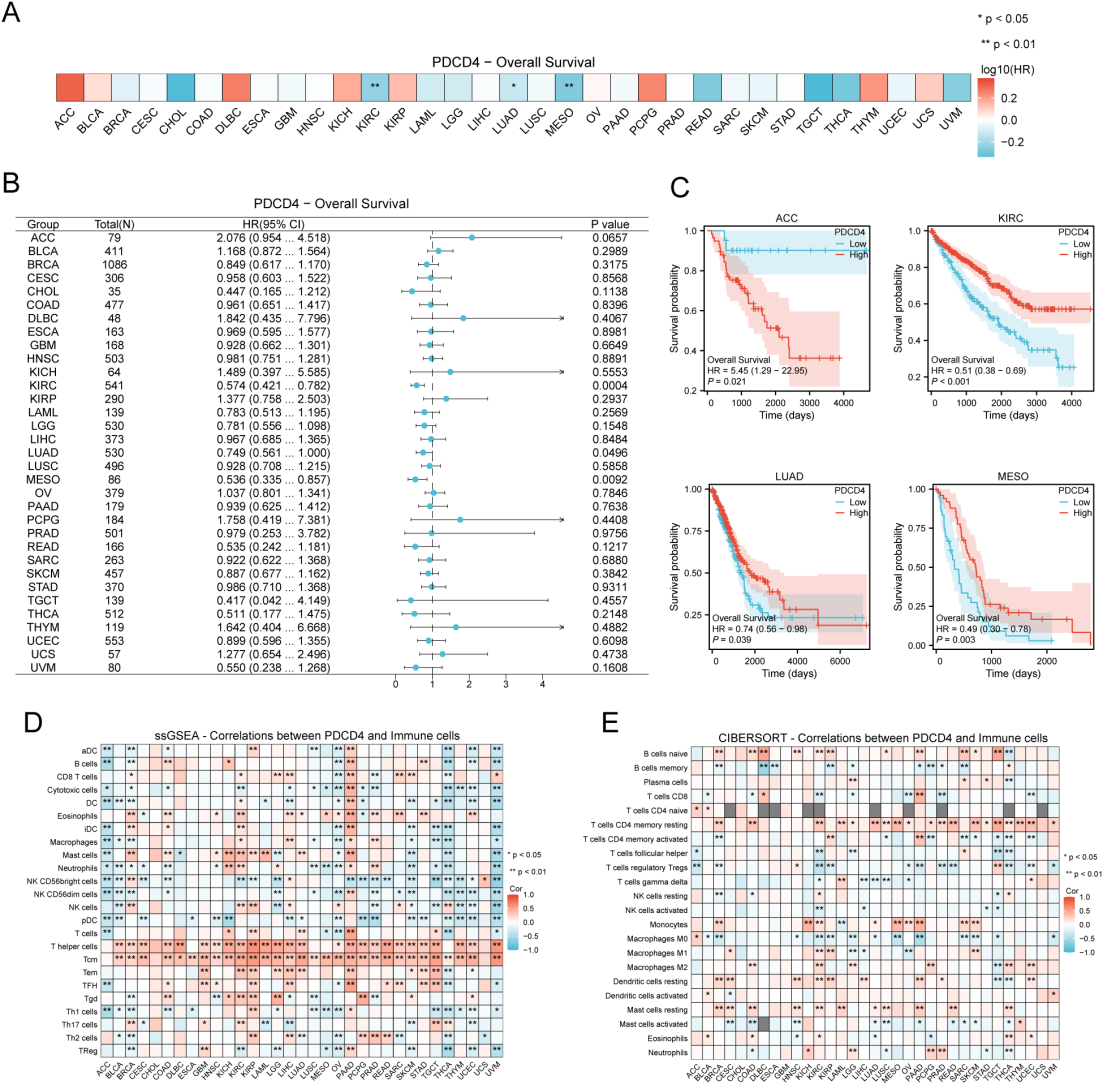
Prognostic value of PDCD4 and its association with immune cell infiltration in pan-cancer analysis. **(A)** Heatmap displaying the OS analysis results for PDCD4 across different cancer types. The color scale represents the log10(hazard ratio, HR), where blue indicates higher survival in the PDCD4 high-expression group and red indicates lower survival. **(B)** Forest plot summarizing the survival analysis of PDCD4 across multiple cancers. The x-axis represents the HR, with points indicating HR values and horizontal lines denoting 95% confidence intervals. **(C)** Kaplan-Meier survival curves illustrating the survival differences between PDCD4 high- and low-expression groups in ACC, KRCC, LADC, and MESO. **(D)** ssGESEAN analysis evaluating the correlation between PDCD4 expression and the infiltration of various immune cell types. Red indicates a positive correlation, while blue indicates a negative correlation. **(E)** CIBERSORT analysis assessing the association between PDCD4 expression and immune cell infiltration, with red indicating a positive correlation and blue indicating a negative correlation. Statistical significance is marked as follows: *p < 0.05; **p < 0.01.

We conducted a thorough pan-cancer research to examine PDCD4 expression patterns, mutational features, prognostic importance, and its possible function in tumor immune regulation because cancer is known to increase the incidence of AF. According to our findings, several forms of cancer have unique patterns of PDCD4 expression. **Figures 8A, B** shows that cholangiocarcinoma (CHOL) and liver hepatocellular carcinoma were highly increased, whereas malignancies including thyroid carcinoma and colon adenocarcinoma were markedly downregulated with respect to PDCD4. In cancers like prostate adenocarcinoma and breast invasive carcinoma, HIGH PDCD4 expression is negatively correlated with important markers of genomic instability, such as tumor mutational burden TMB and microsatellite instability (MSI), according to additional research into the genomic features linked with PDCD4 expression (**Figures 8C, D**). We performed an analysis of PDCD4’s correlation with overall survival (OS) across various cancer types to determine its therapeutic value. An intriguing finding in adrenocortical carcinoma (ACC) is the correlation between high PDCD4 expression and a much worse prognosis, which raises the possibility of an oncogenic function in this cancer. On the flip side, malignancies such kidney renal clear cell carcinoma (KRCC), lung adenocarcinoma (LADC), and mesothelioma (MESO) were shown to have better survival outcomes when PDCD4 levels were higher. This suggests that PDCD4 may have a tumor-suppressing role in these cases (**Figures 9A–C**). **Figures 9D, E** show that PDCD4 may affect tumor-immune interactions in a cancer-specific way, as shown by unique immune cell infiltration patterns linked with PDCD4 expression, as indicated by single-sample gene set enrichment analysis (ssGESEAN) and CIBERSORT analysis. To sum up, our pan-cancer research reveals that PDCD4 is an important molecular actor in several cancers. The fact that PDCD4 influences genomic stability, patient prognosis, and immune modulation implies that it could serve as a link between the biology of cancer and the progression of AF.

## 4 Discussion

Highlighting PDCD4’s significance in inflammation, mitochondrial function, and steroid metabolism, this study revealed critical genes and pathways controlled by it in AF. Endothelial cells were identified as crucial in the AF microenvironment by single-cell RNA sequencing, and differential expression analysis exposed substantial changes in metabolic and endocrine processes. Furthermore, PDCD4 has several functions in cancer, and its dysregulation is associated with tumor growth and an increased risk of AF, so it may be a therapeutic target for both diseases.

Although PDCD4 was first found in apoptotic cells, it is now known that it regulates chronic inflammation and metabolic dysregulation, which play a role in the pathophysiology of numerous diseases. These include cancer, hepatitis, neurological disorders, metabolic diseases, and cardiovascular diseases (25–28). The results of our GSEA enrichment analysis indicated that the activation of signaling pathways associated to lipid metabolism, specifically the PPARα signaling pathway, was dramatically enhanced by PDCD4 deletion. Previous research has found that PDCD4 enhances lipid accumulation in hepatocytes by hindering PPARα-mediated fatty acid oxidation (29), which is in line with our results. It has been demonstrated that the PPAR-α/sirtuin 1/PGC-1α pathway inhibits atrial metabolic remodeling in individuals with AF, which is an essential process in the development of AF (30). Additionally, our GSEA enrichment analysis showed that the PI3K/FGFR1 signaling pathway was significantly activated with PDCD4 overexpression. We found evidence that PDCD4 may have a role in cardiac fibrosis, adding to the literature that suggests it modulates PI3K/AKT signaling to induce cardiomyocyte apoptosis (31). In addition, research has demonstrated that the PPAR-γ/NF-κB pathway is activated by PDCD4, which in turn increases inflammation and fibrosis in mouse atrial myocytes (12). The results of this study highlight the complex function of PDCD4 in AF and point to its possible use as a therapeutic target to address fibrosis, inflammation, and metabolic remodeling in this condition.

We identified eleven important genes related with PDCD4 using differential expression and PPI analysis; of them, PDCD4, SIRT1, and FABP4 showed very significant connections. An NAD+-dependent deacetylase, SIRT1 is essential for controlling cell differentiation, oxidative stress, autophagy, and apoptosis (32). Research has indicated that SIRT1 has the ability to control the activation of PDCD4 through AP-1/decorin signaling (33), indicating that SIRT1 might potentially operate as an intermediary regulator of PDCD4. Furthermore, SIRT1 reduces age-related AF by blocking RIPK1 acetylation and by activating the SIRT1/PGC-1α/FNDC5 axis, it inhibits atrial fibrosis caused by angiotensin II (Ang II) (34). Based on these results, it seems that SIRT1 protects against the advancement of AF via its regulatory connection with PDCD4. We also found that FABP4, a cytosolic protein that is involved in the metabolism and absorption of fatty acids (35), was a major PDCD4-associated gene. By facilitating lipid and fibrotic alterations in heart architecture and Ca2+ dynamics, FABP4 has been recognized as a prognostic indicator for individuals with AF (36, 37). Curiously, research has shown that autophagic proteins drive the unusual secretion of FABP4 in a SIRT1-dependent way (38), and the PPI network has shown a strong relationship between SIRT1 and FABP4. To summarize, our results shed insight on the intricate regulatory network including PDCD4, SIRT1, and FABP4. The fact that SIRT1 regulates PDCD4 and that it is connected to FABP4 implies that there is a coordinated process by which PDCD4 adds to the dysregulation of lipid metabolism and fibrosis in AF.

An AF-specific competing endogenous RNA (cenRNA) network was built by combining interactions between differentially expressed miRNAs, mRNAs, and LncRNAs in order to delve more into the regulatory linkages among the identified important genes. Metastasis Associated Lung Adenocarcinoma Transcript 1 (MET-LAT1) stood out among the differentially expressed LncRNAs. In addition to its role in non-small cell lung cancer prognosis, MET-LAT1 has been linked to cardiovascular diseases (39). According to research, cardiomyocyte apoptosis in AF can be decreased by upregulating Sox-6 expression through the downregulation of miR-499a-5p, which is produced by cardiac fibroblast-derived exosomal MET-LAT1 (40). Furthermore, we found that mmu-miR-429-3p and SIRT1 interact closely, which may indicate that the mmu-miR-429-3p/SIRT1 axis is involved in the pathophysiology of AF. An earlier study found that mmu-miR-429-3p is associated with MET regulation, while another study found that SIRT1 promotes mesenchymal-epithelial transition (MET) via controlling Fra-1 expression (41, 42). This study adds to the growing body of evidence suggesting that the mmu-miR-429-3p/SIRT1 axis may have a role in reducing atrial fibrosis, a major risk factor for AF development. Our cenRNA network study concludes that mRNAs, miRNAs, and LncRNAs all interact intricately to control PDCD4-related gene expression. New regulatory pathways may contribute to fibrosis, inflammation, and metabolic dysregulation in AF, as shown by the involvement of MET-LAT1 and the mmu-miR-429-3p/SIRT1 axis. The results of this study provide hope for the future of AF research into the potential of RNA-based treatment approaches that target the regulatory network focused on PDCD4.

To better understand the roles and activities of these important genes in AF, we used data from single-cell RNA sequencing to investigate the cardiac microenvironment. In AF, we found that the fraction of arterial and venous endothelial cells (ECs) is much lower. This suggests that endothelial dysfunction may contribute to ischemia, thrombosis, and vascular shear stress, which are all associated with the development of AF (43). Furthermore, examination of cell-cell communication revealed robust connections between lymphocytes and artery ECs. When blood vessel ECs in the arteries are under stress, they release cell adhesion molecules including ICAM-1 and VCAM-1. These molecules help monocytes and T cells communicate, and eventually travel to areas of inflammation (44). Abnormal interactions between immune cells and arterial ECs in the chronic inflammatory milieu of AF can activate pro-inflammatory pathways including the NF-κB pathway, which worsens atrial remodeling and fibrosis. Additionally, PDCD4 was shown to be considerably increased in capillary ECs and fibroblast-like ECs in AF, according to a study of the expression patterns of the eleven critical genes across several cell clusters. The results support PDCD4’s hypothesized involvement in AF’s fibrotic processes, which are consistent with its established function in inducing fibrosis (12). This study provides more evidence that PDCD4 is involved in the pathophysiology of AF, which has important consequences for its function in endothelial dysfunction, chronic inflammation, and atrial fibrosis.

At last, we discovered medicinal compounds that may target the eleven critical genes identified in this study. In particular, trichostatin A (TSA), a histone deacetylase inhibitor that targets ATAD5, has shown promise in reducing chronic inflammation and fibrosis in other disorders such as inflammatory bowel disease and hepatic ischemia-reperfusion damage (45, 46). TSA exerts its effects by modulating histone acetylation, which can influence gene expression related to inflammation and fibrosis. Given the role of inflammation and fibrosis in AF pathophysiology, it is plausible that TSA could reduce these pathological processes in AF as well.

Although the direct effects of TSA on AF have not been thoroughly studied, its potential to modify the inflammatory and fibrotic remodeling in AF provides a compelling rationale for further investigation. Future studies are necessary to examine whether TSA can target PDCD4-related pathways in AF, particularly those involved in fibrosis and endothelial dysfunction. Additionally, understanding the molecular mechanisms through which TSA may influence PDCD4 expression and related signaling networks could enhance its translational relevance and therapeutic potential for AF.

Due to the same inflammatory and metabolic pathways between cancer and AF, deregulation of PDCD4 in certain malignancies might increase the risk of AF (12, 44). Our results imply a more complex function for PDCD4 across various malignancies, challenging its long-standing reputation as a tumor suppressor (45). In contrast to its protective function in KRCC and LADC, we found that overexpression of PDCD4 is linked to a poor prognosis in patients with ACC. Research on PDCD4 in ACC is still in its early stages, but its immunomodulatory activities may be the cause of its carcinogenic potential in this setting. While PDCD4 seems to increase immune cell infiltration in most malignancies, our data show that it suppresses it in ACC. The relationship between PDCD4 and poorer outcomes in these patients may be explained by its involvement in modifying the tumor immune milieu. This might be because immune evasion is a hallmark of ACC and contributes to its aggressive nature. Another hallmark of ACC is chronic glucocorticoid excess, which is known to cause structural and functional alterations in the heart that provide a substrate that promotes arrhythmias. The results point to a possible biological connection between immune dysregulation, PDCD4, and the likelihood of AF in ACC patients. Therefore, it seems that targeting PDCD4 might be a great way to treat AF and ACC. In addition to reducing inflammation and remodeling caused by AF, restoring immune surveillance in ACC may be possible by modulating PDCD4 expression. Patients at risk of both illnesses may find new treatment options if future research investigates the viability of PDCD4-targeted medicines in this dual scenario.

Recent advances in cancer immunotherapy provide important context for interpreting our findings. For example, circulating biomarkers such as soluble PD-L1 and cytokine profiles have shown promise in predicting responses to immune checkpoint inhibitors in melanoma, highlighting the clinical relevance of immune-related gene signatures similar to those identified in our study (47). Additionally, emerging CAR T-cell therapies are being designed to overcome metabolic challenges in solid tumors by enhancing mitochondrial function and resistance to tumor microenvironment stressors (48), which aligns with the metabolic pathways linked to PDCD4 observed in our analysis.

Beyond this, growing insights into the PD-1/PD-L1 signaling axis in cancers like gastric cancer suggest that combination immunotherapies and stratification based on tumor microenvironment characteristics may improve treatment outcomes (49). Moreover, novel immunomodulatory approaches that target communication between tumor cells and tumor-associated macrophages—such as interference via small extracellular vesicles—offer promising strategies to reshape the tumor microenvironment and enhance anti-tumor immunity (50). These developments emphasize the need to consider both cancer cells and their surrounding stromal and immune context in therapeutic design.

## 5 Conclusion

This study provides an in-depth analysis of PDCD4’s regulatory roles in AF, highlighting key genes, pathways, and potential therapeutic targets. By integrating transcriptomic, cenRNA, and single-cell RNA sequencing data, we elucidated the complex molecular mechanisms involved in AF progression. While these findings offer valuable insights, further validation through larger patient cohorts and experimental models is needed. Additionally, functional studies to confirm the roles of the identified genes and pathways are crucial. Future research should continue to explore the therapeutic potential of targeting these regulatory networks, aiming to improve treatment and prevention strategies for AF.

## 6 Limitation and future perspectives

This study elucidates the regulatory role of PDCD4 in AF and identifies potential therapeutic targets through multi-omics analysis and molecular docking. However, several limitations should be acknowledged. First, the predicted drug–target interactions lack experimental validation, which limits the clinical translatability of these findings. Second, transcriptomic analyses were primarily based on PBMCs and public datasets such as TCGA, which are subject to inherent technical and biological biases, including tumor heterogeneity, sample purity, and batch effects. These factors may confound gene expression interpretation and do not fully capture tissue-specific or spatial dynamics. Moreover, bulk RNA-seq data cannot distinguish expression derived from specific cell types within complex microenvironments, limiting insights into the precise cellular origins and functional relevance of PDCD4 expression. Importantly, correlation does not imply causation; elevated gene expression may reflect disease progression or immune response rather than a direct causal role. In future studies, *in vitro* and *in vivo* experiments, including validation in atrial tissues and functional assays of candidate compounds, are needed to strengthen the conclusions and explore therapeutic applicability. To better assess the functional roles of PDCD4 and its associated hub genes, genome-wide CRISPR screening combined with pharmacogenomic profiling—as demonstrated in recent drug resistance research—may offer a powerful strategy to uncover actionable targets. Additionally, incorporating patient-derived xenograft (PDX) models in future research could further enhance translational relevance, as these models preserve molecular and microenvironmental features of human tumors and allow *in vivo* validation of immunological and therapeutic mechanisms identified through computational analyses.

## Data Availability

The datasets presented in this study can be found in online repositories. The names of the repository/repositories and accession number(s) can be found in the article/**Supplementary Material**.

## Supplementary material

Supplementary Figure 1 mRNA Expression Levels and Sample Distribution Analysis. Boxplots displaying mRNA expression levels in the control, PDCD4-KO, and PDCD4-OE groups.

Supplementary Figure 2 Differential Expression and Distribution of LncRNAs and miRNAs **(A)** Boxplots displaying LNC-RNA expression levels in the control, PDCD4-KO, and PDCD4-OE groups. **(B)** PC plot showing the clustering of LNC-RNA samples in the control, PDCD4-KO, and PDCD4-OE groups. **(C)** Volcano plot illustrating differentially expressed LncRNAs in the PDCD4-KO group compared to the control group, highlighting the top 10 upregulated and downregulated LncRNAs. **(D)** Heatmap showing the expression levels of the top 10 upregulated and downregulated LncRNAs in the PDCD4-KO group compared to the control group. **(E)** Volcano plot illustrating differentially expressed LncRNAs in the PDCD4-OE group compared to the control group, highlighting the top 10 upregulated and downregulated LncRNAs. **(F)** Heatmap showing the expression levels of the top 10 upregulated and downregulated LncRNAs in the PDCD4-OE group compared to the control group. **(G)** Boxplots displaying miRNA expression levels in the control, PDCD4-KO, and PDCD4-OE groups. **(H)** PC plot showing the clustering of miRNA samples in the control, PDCD4-KO, and PDCD4-OE groups. **(I)** Volcano plot illustrating differentially expressed miRNAs in the PDCD4-KO group compared to the control group, highlighting the top 10 upregulated and downregulated miRNAs. **(J)** Heatmap showing the expression levels of the top 10 upregulated and downregulated miRNAs in the PDCD4-KO group compared to the control group. **(K)** Volcano plot illustrating differentially expressed miRNAs in the PDCD4-OE group compared to the control group, highlighting the top 10 upregulated and downregulated miRNAs. **(L)** Heatmap showing the expression levels of the top 10 upregulated and downregulated miRNAs in the PDCD4-OE group compared to the control group.

Supplementary Figure 3 Quality Control, Variable Gene Analysis, and Cell Clustering in SCRNA-SEQ Data **(A, B)** Violin plots displaying the number of detected genes, total mRNA molecules, and the proportion of mitochondrial gene expression before and after quality control. **(C)** Volcano plot showing highly variable genes, with the top 10 genes labeled. **(D, E)** PC plots showing dimensionality reduction of highly variable genes. **(F)** UMAP plot visualizing the clustering of cells into 21 subpopulations. **(G)** Bubble plot showing the annotation of 21 cell clusters based on marker genes. **(H)** Bar chart comparing the proportion of different cell clusters between AF and control samples. **(I)** Heatmap displaying the cell-cell communication analysis of eight identified cell types.

Supplementary Figure 4 Expression of Key Genes in Different Cell Clusters **(A-J)** UMAP plots showing the expression levels of 11 key genes ("Sirt1, PDCD4, Fabp4, Hsd3b2, Rgs1, Rgs13, Atad5, Hltf, Abi2, Wasl, Hsd3b3") across various cell clusters identified in the SCRNA-SEQ analysis.
