# Correction: Targeting PDCD4 in cancer and atrial fibrillation: mechanistic insights from integrated multi-omics and single-cell analysis

**DOI:** 10.3389/fonc.2025.1756715

**Published:** 2025-12-15

**Authors:** Juledezi Hailati, Zhiqiang Liu, Lei Zhang, Muhuyati Wulasihan

**Affiliations:** Cardiovascular Disease Center, The First Affiliated Hospital of Xinjiang Medical University, Urumqi, China

**Keywords:** atrial fibrillation, PDCD4, transcriptome sequencing, single-cell RNA sequencing, pan-cancer analysis

The reference for marker gene identification was erroneously written as “PMlD.3727811”. It should be “PMlD: 37440641”.

The original version of this article has been updated.

