# Supplementary figures and images for: Targeting PDCD4 in cancer and atrial fibrillation: mechanistic insights from integrated multi-omics and single-cell analysis
Source: Front Oncol. 2025 Jul 22;15:1593815. doi: 10.3389/fonc.2025.1593815 (PMC12321552; doi:10.3389/fonc.2025.1593815)

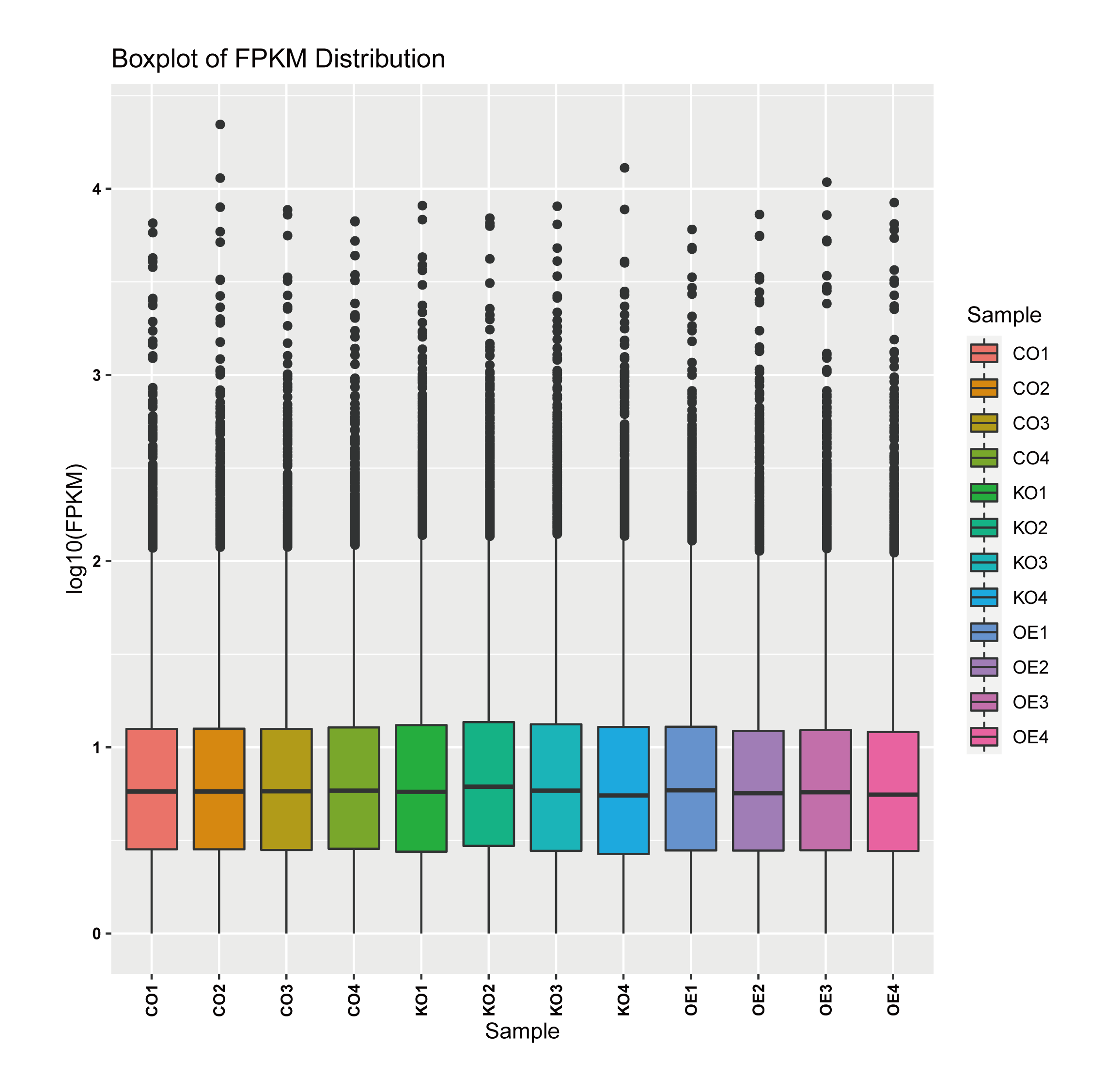

Supplement: Supplementary Figure 1 — mRNA Expression Levels and Sample Distribution Analysis. Boxplots displaying mRNA expression levels in the control, PDCD4-KO, and PDCD4-OE groups. [file Image1.tif]

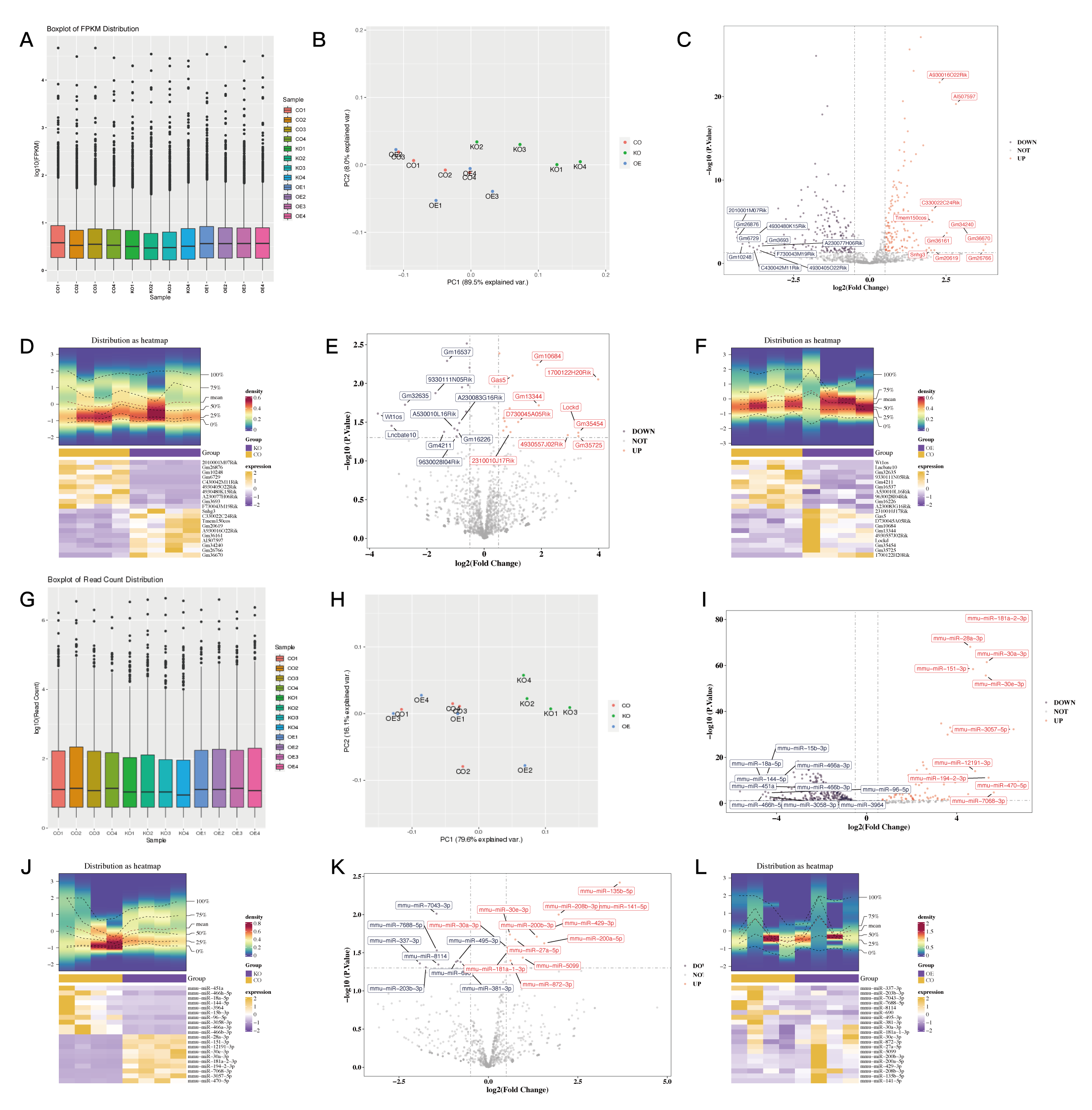

Supplement: Supplementary Figure 2 — Differential Expression and Distribution of LncRNAs and miRNAs (A) Boxplots displaying LNC-RNA expression levels in the control, PDCD4-KO, and PDCD4-OE groups. (B) PC plot showing the clustering of LNC-RNA samples in the control, PDCD4-KO, and PDCD4-OE groups. (C) Volcano plot illustrating differentially expressed LncRNAs in the PDCD4-KO group compared to the control group, highlighting the top 10 upregulated and downregulated LncRNAs. (D) Heatmap showing the expression levels of the top 10 upregulated and downregulated LncRNAs in the PDCD4-KO group compared to the control group. (E) Volcano plot illustrating differentially expressed LncRNAs in the PDCD4-OE group compared to the control group, highlighting the top 10 upregulated and downregulated LncRNAs. (F) Heatmap showing the expression levels of the top 10 upregulated and downregulated LncRNAs in the PDCD4-OE group compared to the control group. (G) Boxplots displaying miRNA expression levels in the control, PDCD4-KO, and PDCD4-OE groups. (H) PC plot showing the clustering of miRNA samples in the control, PDCD4-KO, and PDCD4-OE groups. (I) Volcano plot illustrating differentially expressed miRNAs in the PDCD4-KO group compared to the control group, highlighting the top 10 upregulated and downregulated miRNAs. (J) Heatmap showing the expression levels of the top 10 upregulated and downregulated miRNAs in the PDCD4-KO group compared to the control group. (K) Volcano plot illustrating differentially expressed miRNAs in the PDCD4-OE group compared to the control group, highlighting the top 10 upregulated and downregulated miRNAs. (L) Heatmap showing the expression levels of the top 10 upregulated and downregulated miRNAs in the PDCD4-OE group compared to the control group. [file Image2.tif]

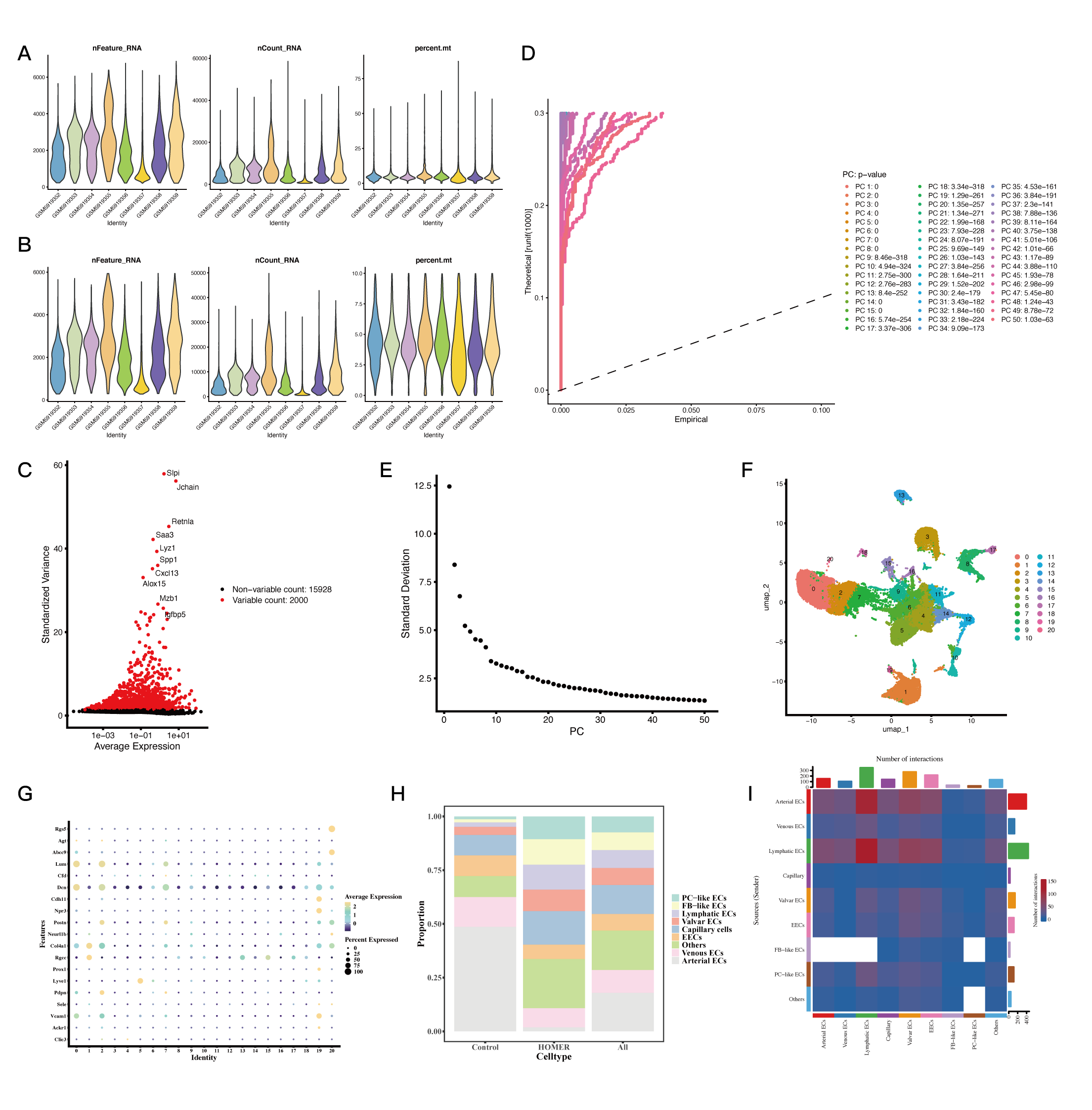

Supplement: Supplementary Figure 3 — Quality Control, Variable Gene Analysis, and Cell Clustering in SCRNA-SEQ Data (A, B) Violin plots displaying the number of detected genes, total mRNA molecules, and the proportion of mitochondrial gene expression before and after quality control. (C) Volcano plot showing highly variable genes, with the top 10 genes labeled. (D, E) PC plots showing dimensionality reduction of highly variable genes. (F) UMAP plot visualizing the clustering of cells into 21 subpopulations. (G) Bubble plot showing the annotation of 21 cell clusters based on marker genes. (H) Bar chart comparing the proportion of different cell clusters between AF and control samples. (I) Heatmap displaying the cell-cell communication analysis of eight identified cell types. [file Image3.tif]

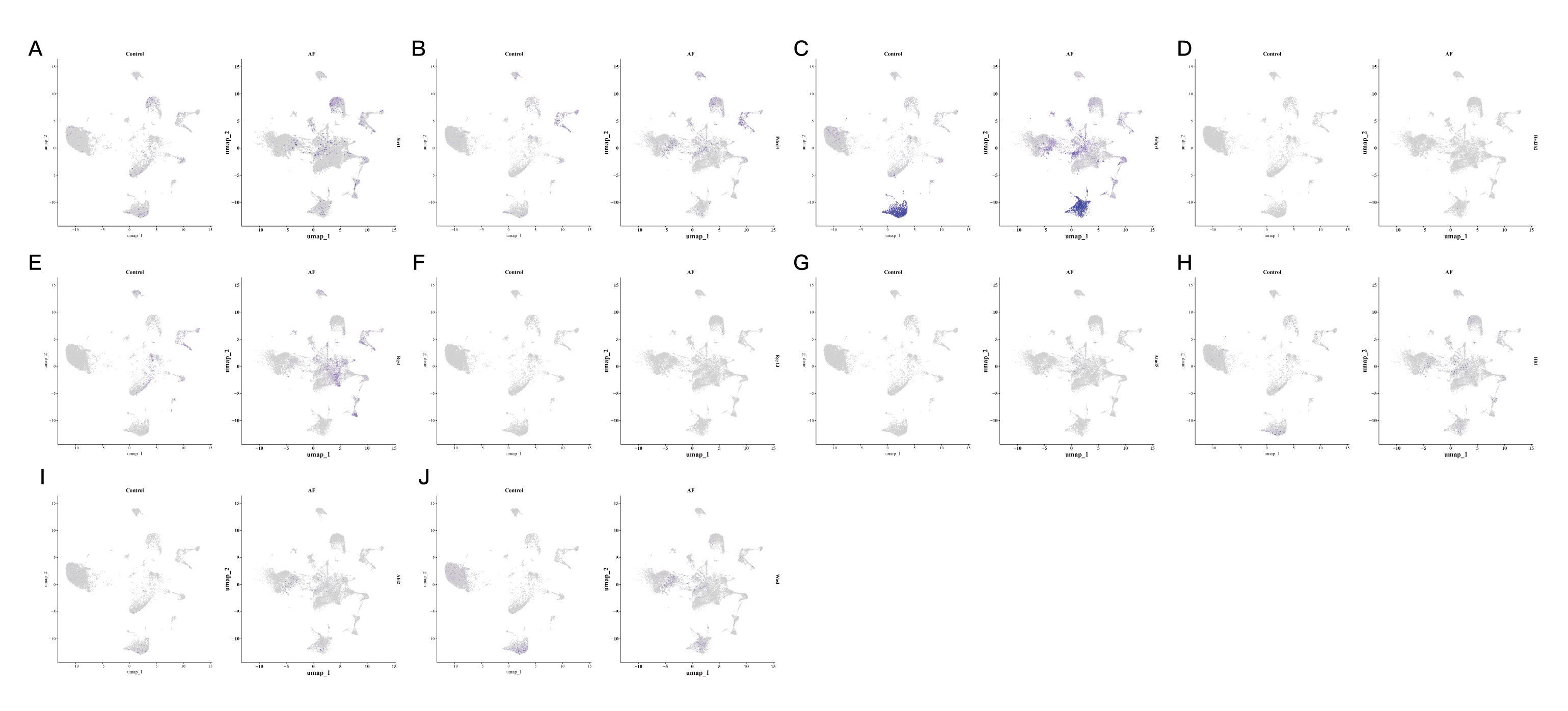

Supplement: Supplementary Figure 4 — Expression of Key Genes in Different Cell Clusters (A-J) UMAP plots showing the expression levels of 11 key genes ("Sirt1, PDCD4, Fabp4, Hsd3b2, Rgs1, Rgs13, Atad5, Hltf, Abi2, Wasl, Hsd3b3") across various cell clusters identified in the SCRNA-SEQ analysis. [file Image4.tif]
