# Supplementary material for: Targeting PDCD4 in cancer and atrial fibrillation: mechanistic insights from integrated multi-omics and single-cell analysis
Source: Front Oncol. 2025 Jul 22;15:1593815. doi: 10.3389/fonc.2025.1593815 (PMC12321552; doi:10.3389/fonc.2025.1593815)
Supplement: Supplementary file 5 [file Table1.docx]

**Supplementary table 1. A list of primers used in this study.**

| Gene | Forward sequence (5’ to 3’) | Reverse sequence (5’ to 3’) |
| --- | --- | --- |
| GAPDH | GGAGCGAGATCCCTCCAAAAT | GGCTGTTGTCATACTTCTCATGG |
| PDCD4 | GCAAAAAGGCGACTAAGGAAAAA | TAAGGGCGTCACTCCCACT |
